# Innovation in Osteogenesis Activation: Role of Marine-Derived Materials in Bone Regeneration

**DOI:** 10.3390/cimb47030175

**Published:** 2025-03-07

**Authors:** Maria Giovanna Rizzo, Marilena Briglia, Vincenzo Zammuto, Dario Morganti, Caterina Faggio, Federica Impellitteri, Cristiana Roberta Multisanti, Adriana Carol Eleonora Graziano

**Affiliations:** 1 Department of Chemical, Biological, Pharmaceutical and Environmental Sciences, University of Messina, Viale Ferdinando Stagno d’Alcontres, 31, 98166 Messina, Italy; vzammuto@unime.it; 2 Department of Medicine and Surgery, “Kore” University of Enna, 94100 Enna, Italy; marilena.briglia@unikore.it (M.B.); adriana.graziano@unikore.it (A.C.E.G.); 3 Consiglio Nazionale delle Ricerche DSFTM, Department of Physical Sciences and Technologies of Matter, Piazzale Aldo Moro, 7, 00185 Roma, Italy; 4 Department of Eco-Sustainable Marine Biotechnology, Stazione Zoologica Anton Dohrn, 80122 Naples, Italy; 5 Department of Veterinary Sciences, University of Messina, Viale Giovanni Palatucci, 98168 Messina, Italy; federica.impellitteri@studenti.unime.it (F.I.); cristiana.multisanti@studenti.unime.it (C.R.M.)

**Keywords:** bone tissue regeneration, marine biomaterials, eco-physiology, blue biotechnologies, molecular pathways, marine ecology

## Abstract

Marine-derived biomaterials are emerging as promising candidates for tissue regeneration due to their sustainability, biocompatibility, bioactivity, and unique chemical structure. This review provides an overview of different marine-derived inorganic and organic materials, such as calcium carbonate, magnesium salts, silica, polysaccharides, bioactive peptides, and lipid-based compounds, and their effects in promoting osteogenesis. Specifically, the osteoinductive, osteoconductive, and osteointegrative activities of traditional and innovative materials that influence key molecular pathways such as BMP/Smad and Wnt/β-catenin signaling underlying bone formation will be evaluated. This review also prospects innovative approaches, i.e., phage display technology, to optimize marine-derived peptides for targeted bone regeneration. In the context of innovative and sustainable materials, this review suggests some interesting applications of unusual materials able to overcome the limitations of conventional ones and stimulate cellular regeneration of bone tissue by activating specific molecular pathways.

## 1. Introduction

Osteogenesis, the process responsible for the formation of new bone tissue, plays a crucial role in bone development, growth, and repair and is essential in responses to bone injury. However, in some clinical scenarios, such as fractures or in the case of a bone tumor, this process may be limited. Under physiological conditions, bone has the intrinsic capacity to regenerate itself. This process involves the activation of intramembranous or endochondral ossification [1]. Intramembranous ossification occurs mainly in flat bones, where mesenchymal stem cells differentiate directly into osteoblasts, leading to bone formation without a cartilaginous intermediary. Indeed, previous studies show that intramembranous ossification depends directly on the differentiation of mesenchymal tissue to bone tissue and is associated with osteoblast-induced remodeling [2]. On the other hand, endochondral ossification is predominantly in long bones, where a cartilage template is first formed and then replaced by bone tissue. This process requires the migration of mesenchymal cells to form pre-cartilaginous condensations after cellular differentiation into chondrocytes, which deposit cartilaginous matrix, followed by osteoblast-mediated ossification [3,4].

Although these two pathways are usually described in bone development based on the cellular and molecular mechanisms involved, they are reactivated during fracture repair, suggesting great dynamism between skeletal precursors and differentiated cell lineages [5].

In recent years, researchers in biomedical technologies and regenerative medicine have focused on developing tools and procedures that leverage the body’s intrinsic self-repair mechanisms to facilitate the regeneration of large and complex bone defects. By integrating tissue engineering, biomaterials science, stem cell therapy, and bioactive molecule delivery, these approaches aim to restore normal bone function effectively [6].

In the field of bone regeneration, the application of biomaterials is gaining more and more interest and numerous solutions have been proposed. According to their origin, materials are distinguished into autografts if derived from patients themselves, allografts derived from different donors of the same species, xenografts grafted from different species, and alloplasts as synthetic biomaterials [7,8]. Scaffolds provided by materials seem to promote the reconstruction of damaged tissues, restoring the lost anatomy and function. Particularly in tissue engineering, the choice of scaffold is made considering highly sophisticated materials and structures designed to initially promote cell adhesion and proliferation [9]. This process ensures the deposition of new bone tissue, which can replace the artificial implant. Therefore, the key role of biomaterials is to provide mechanical support for the cells involved in the formation and regeneration processes and to ensure three-dimensional temporary guidance. Originally, biologically inert materials with the advantage of reduced immune activation response in the host organism were used in the production of bone grafts. As recently reviewed by Girón et al. [10], the application of alloplastic biomaterials has the advantages of easy handling, great availability of shapes and sizes, and a large variety of resources for its production. Actually, traditional bone grafts and synthetic materials have limitations, such as donor site morbidity, immune rejection, suboptimal integration, and mechanical incompatibility [11]. Concerning treatment methods to improve performance in clinical applications, several types of approaches have been adopted: (i) customizing the surface architecture of the synthetic bone graft (BGS); (ii) enriching the BGS with bioactive molecules such as growth factors, peptides or small molecules targeting bone precursor cells; and (iii) cell-based approaches involving progenitor cells, alone or in combination with active molecules, which can be injected or seeded onto the BGS to improve delivery [12].

Over the past thirty years, the trend has been from bioinert materials to bioactive materials. An excellent bioactive material, proposed as a bone substitute, should induce osteogenesis, osteoinduction, and osteoconduction. These three phenomena are biologically interrelated but not equal: to briefly summarize the osteoinductive properties of a biomaterial aid to form a new bone, the osteoconductive ones give structural support, while osseointegration ensures the bone anchorage over a long period and during functional loading.

Consequently, there is a need to discover and use new innovative materials that can stimulate osteogenesis and promote seamless integration with host tissues. In this context, biomaterials of marine origin have attracted considerable attention due to their unique biochemical compositions, biocompatibility, and bioactivity, characteristics that make them efficient materials for bone tissue engineering applications [13]. The major advance of marine biomaterials resides in their natural source: the marine environment, characterized by its vast biodiversity, offers a series of bioactive compounds and materials that can be exploited for biomedical applications.

For the above-mentioned reasons, this review article aims to provide a comprehensive overview of the current advances in the use of marine-derived biomaterials for bone regeneration, starting with an overview of the main characteristics that distinguish a biomaterial, in association with the biological and molecular processes of bone regeneration. Focus has been placed on the functional role of marine biomaterials in bone repair. The aim is to define their chemical profile, their origin, and their role in osseointegration, osteoconduction, and osteoinduction. Furthermore, we critically describe some innovative techniques that could enable the identification of biomaterials of marine origin in the context of their application in bone regenerative medicine. Overall, this study aims to revise the unique properties of marine biomaterials to debate their potential application to overcome the limitations of current therapies.

## 2. Biological Profile and Activity of Biomaterials for Bone Regeneration

The emulation and induction of bone regenerative physiological processes are very challenging in the context of regenerative medicine. Following trauma or fracture, a myriad of biological processes occur that promote tissue development, homeostasis, and repair. In the human body, the process mimics embryonic bone development and could be classified as intramembranous or endochondral, as reported in the introductory section [1].

**Figure 1:**
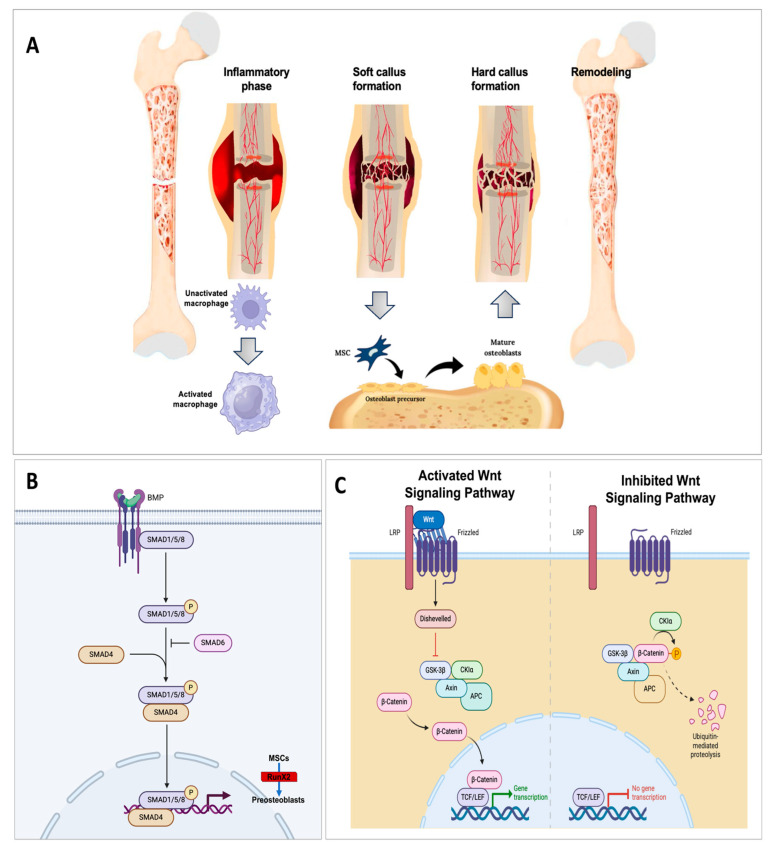
(**A**) Summary of healing stages following a bone fracture. MSC: mesenchymal stem cells. (**B**) Overview of the bone morphogenetic proteins (BMP) signaling pathway which promotes the commitment of MSCs to osteogenic lineages. BMP binds to a pre-assembled receptor complex composed of BMPRI, BMPRII, and BMPRII phosphorylates (specified as P) BMPRI, which then induces intracellular phosphorylation of small mother against decapentaplegic (Smad) Smad1/5/8. Subsequently, the phosphorylated Smad1/5/8 forms a complex with Smad4 and enters the nucleus to regulate transcription of the target gene Runt-related transcription factor 2 (RUNX2), a key transcription factor associated with osteoblast differentiation. (**C**) Wnt/β-catenin pathway, which regulates osteoblast proliferation and differentiation. Activated Wnt signaling pathway: Wnt binds the receptor frizzled and the co-receptor low-density lipoprotein receptor-related protein (LRP5/6) and activates the cytoplasmic protein disheveled (DVL) that induces aggregation of the complex (AXIN: Anti-Neurexin. GSK3β: glycogen synthase kinase-3β; CK1: casein kinase 1; APC: *Adenomatous polyposis* coli gene) and GSK3β suppression. Stabilized β-catenin moves into the nucleus and binds to TCF/LEF transcription factors to lead to the target gene transcription. Inhibited Wnt signaling pathway: without Wnt, the β-catenin destruction complex (AXIN, CK1α, GSK3β, and APC) is activated, and phosphorylated β-catenin undergoes ubiquitin proteasomal degradation. (Partially created with BioRender: Toronto, ON, Canada, www.biorender.com, accessed on 10 January 2025.)

The physiological process of bone repair is characterized by four main phases: (i) the inflammatory phase; (ii) the soft callus formation; (iii) the hard callus formation; and (iv) the remodeling phase [14] (Figure 1A). In the first phase (the inflammatory phase), platelets and immune cells (macrophages, neutrophils) release pro-inflammatory cytokines such as tumor necrosis factor-alpha (TNF-α) and interleukin (IL-1, IL-6) to recruit mesenchymal stem cells (MSCs). Moreover, macrophages play the main role in removing necrotic debris and stimulating angiogenesis by releasing vascular endothelial growth factor (VEGF).

Soft callus formation is typical of the endochondral pathway: MSCs differentiate into chondrocytes, forming a soft cartilaginous callus that fills the fracture space. At a molecular level, the inductive factors of chondrogenesis and osteoblast recruitment are the bone morphogenetic proteins (BMPs), particularly BMP-2, BMP-4, and BMP-7 [15], which promote the commitment of MSCs to osteogenic lineages by the BMP/Smad pathway (Figure 1B).

Then, the hard callus formation stage starts with the chondrocytes that undergo hypertrophy and mineralization, producing a hard callus composed of woven bone. Osteoblasts differentiate from MSCs and secrete osteoid matrix, which later mineralizes with calcium and phosphate deposition. The molecular orchestration of this phase is made by the homeostatic balancing of the Wnt/β-catenin signaling pathway that promotes the proliferation and differentiation of osteoblasts. The activation of the Wnt/β-catenin pathway occurs when Wnt ligands bind to Frizzled receptors and to the co-receptor called low-density lipoprotein receptor-bound proteins (LRP5/6). This event leads to the stabilization and accumulation of β-catenin in the cytoplasm, followed by β-catenin translocation into the nucleus, where it interacts with transcription factors to upregulate the expression of osteogenic genes (Runx2, Osterix, Collagen type I) and promote the differentiation of MSCs into osteoblasts (Figure 1C).

In the remodeling stage, osteoclasts resorb the excess of callus, while osteoblasts deposit organized collagen fibers and minerals. This step requires the regulation of osteoclast differentiation and activation by the RANK/RANKL/osteoprotegerin (OPG) pathway [16,17]. The Wnt/β-catenin pathway and the BMP/Smad pathways work cooperatively to improve osteoblast differentiation, bone matrix deposition, and mineralization [18]. Following the described events, bone fractures heal by themselves. The dysregulation of these processes can compromise bone integrity resulting in a slower or nonresolution of a fracture. Especially in cases of critical-sized bone defects, the need for innovative approaches to support and improve osteogenesis has been reported [19].

In this context, the search for efficient biomaterials that can be applied as support for bone regeneration is gaining increasing interest from the scientific community [20].

An excellent bioactive material that could be proposed as a bone substitute must have optimal biocompatibility, osteointegrative, osteoconductive, and osteoinductive properties [21,22]. As reported by Albrektsson and Johansson [23], the terms osteoinduction, osteoconduction, and osteointegration should be applied correctly and seem to have academic significance, especially in medical research.

Osteoinduction is the ability of a tissue to induce the differentiation of undifferentiated and pluripotent mesenchymal cells from the receptor site, in osteogenic elements (osteoblasts), stimulating the bone neogenesis at both the graft and the recipient site. Cell differentiation is made possible by the release of biomolecules from the graft. To date, demineralized bone [24], BMPs, fibroblast growth factor-2, and insulin-like growth factor-I have been reported as osteoinductive agents. Therefore, the osteoinduction process mediated by biomaterials mimics the physiological phases that begin immediately after injury. In this context, biomaterials could act as osteoinducers thanks to their physicochemical and/or structural properties, such as micro- and nano-structures that can support interaction with BMPs, or thanks to the release of inorganic ions (as in the case of calcium phosphate ceramics) that represent a direct trigger of osteogenic differentiation [25].

Furthermore, biomaterials have also been applied as delivery systems for osteoinductive agents in bone tissue engineering [26,27]. This is the case with hydrogel systems that can be physically or chemically bound to osteoinductive agents, such as alginate microspheres and a 3D-printed scaffold coated with polydopamine [28].

Osteoconduction is the ability of the graft to create structural support for bone formation [29]. It occurs when the graft material forms a three-dimensional network that acts as a scaffold to guide the growth of osteoblasts and their precursors from the walls of the bone defect. This is possible due to the vascularization and occupation of the graft by progenitor cells from the recipient tissue (creeping substitution) [30]. The profile of a biomaterial classified as an osteoconductor lies in its chemical composition, surface properties, and structural geometry. To date, osteoconductivity has been reported for calcium-phosphate-based, porous materials prepared from Bioglass powder [31,32]. Furthermore, it has been reported that the metal surface coating supports osteoconductivity, as in the case of titanium [32,33].

Osseointegration refers to the ability of a biomaterial or implant to integrate into its environment [34]. This process depends on the tissue’s response to a foreign material and, in particular, it is related to biocompatibility at the biomaterial–cell interface. Synthetic and metallic implants present an interface with the host tissue; the formation of a homogeneous interface must be achieved by chemical or physical modification of surfaces [35,36].

Under the described background, the search for an ideal material, to be applied alone or in combination, has gained attention, leading up to the exploration of marine derivatives as a new potential source of biomaterials in the bone healing field.

## 3. Marine-Derived Biomaterials: Chemical Profile and Biological Activity

A rich biodiversity characterizes marine environments. For this reason, it offers a series of bioactive compounds or materials that can be exploited for biomedical applications.

Specifically, for bone repair, the main advance of marine-derived materials resides in their chemical composition, which could drive a biological response that stimulates and/or potentates the biological process of osteogenesis [37].

Marine organisms, including fish, jellyfish, corals, sponges, and various invertebrates, are recognized as significant sources of marine bioactive chemical compounds (minerals, peptides, and lipids) due to their numerous advantages over traditional sources [38].

Marine-derived biomaterials have garnered significant attention in recent years due to their unique biochemical compositions, metabolic compatibility with human systems, and bioactivity, which are advantageous for bone tissue engineering applications [39].

**Figure 2:**
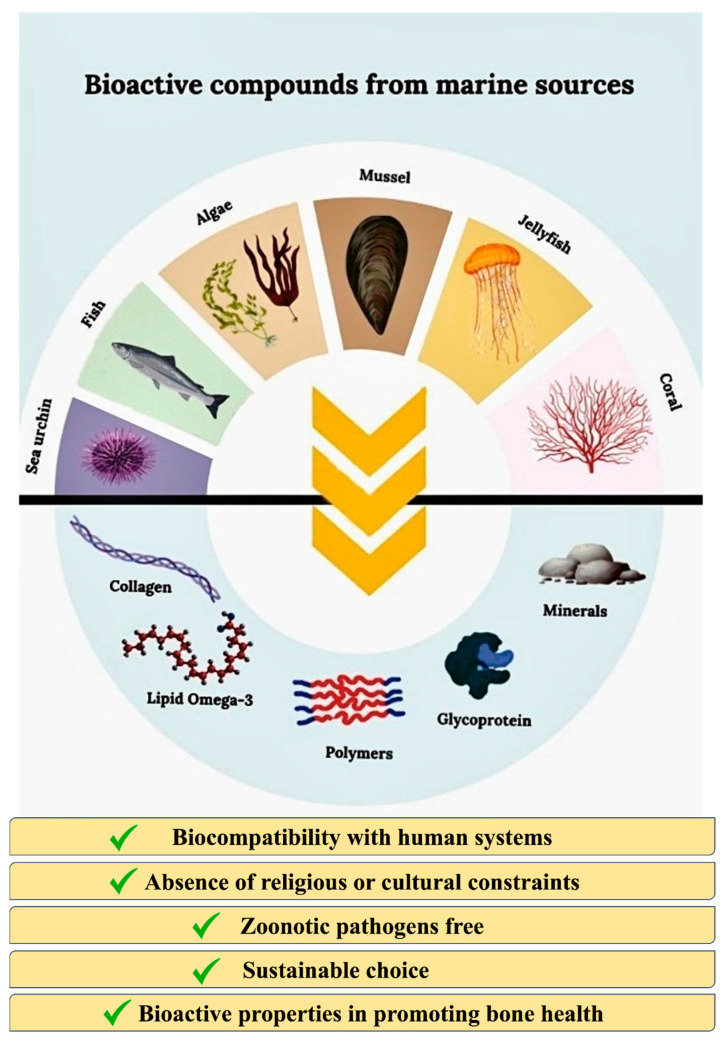
Schematic representation of main sources of marine bioactive compounds (collagen, lipid Omega-3, polymers, glycoproteins, and minerals) obtained by sea urchins, fish, mussels, algae, jellyfish, and coral and their properties to promote bone health. (Created partially with BioRender.com. Toronto, ON, Canada, www.biorender.com, accessed on 29 January 2025.)

Moreover, they could be favored in the choice for the absence of religious or cultural constraints, the freedom from zoonotic pathogens, and they also offer a sustainable approach to reducing waste and promoting the circular economy, considering that several biomaterials can be derived or extracted from by-products of the alimentary industry (Figure 2). Indeed, to date, there has been a growing trend to use byproducts of the fishing industry, such as crustacean, algae, and aquatic plant derivatives, in experimental projects for the production of food, feed, pharmaceuticals, and cosmetics, as well as to produce biomaterials and biofuels and to improve water treatment efficiency [40]. In relation to the different natures of biomaterials, several extraction techniques have been implemented, including hydrolyzed by chemical and enzymatic methods. Moreover, several studies report that to improve extraction efficiency, the above-mentioned approaches could be combined with physical methods like microwave, ultrasound, and pulsed electric field processing [41].

This innovation in the use of waste products represents an initial and sustainable practice to reduce the impact of waste produced by the fishing industry. Indeed, in general, overexploitation of marine resources can significantly alter the balance of ecosystems, leading to loss of biodiversity, reduced resilience, and the spread of invasive species. In order to mitigate these impacts, it is crucial to adopt a sustainable approach that integrates scientific innovation with current ethical and environmental standards, ensuring a fair use of resources. Overexploitation of marine resources can significantly alter the balance of ecosystems, leading to loss of biodiversity, reduced resilience, and the spread of invasive species. In order to mitigate these impacts, it is crucial to adopt a sustainable approach that integrates scientific innovation with current ethical and environmental standards, ensuring fair use of resources and equitable distribution of benefits [42].

All these materials play a key role in several mechanisms such as regulating cellular responses, activating osteogenesis and osteoclastogenesis pathways, and modulating self-assembly by patient cells as intelligent devices with tissue-specific detection capabilities [43].

Marine-derived materials have both inorganic and organic components acting as bioactive compounds, and materials of marine origin have been engineered into functional scaffolds that can adapt and evolve in a favorable environment during the regeneration process. Both aspects are described in the next sections.

### 3.1. Marine-Derived Inorganic Materials

#### 3.1.1. Calcium Carbonate

Calcium carbonate (CaCO_3_) is a common compound found in marine and aquatic environments, sourced from various biological origins (such as fish, mollusks, corals, and calcifying organisms), as well as abiotic processes (like precipitation). These sources are crucial in regulating the global carbon cycle and influencing marine ecosystem dynamics. Of particular note is the amorphous form of calcium carbonate found in marine settings, which demonstrates exceptional stability, making it an ideal material for biomedical applications [44,45].

Research has shown that marine calcium carbonate has promising osteoconductive properties, and it can improve the osteoconductive properties of hydroxyapatite and calcium phosphate-based ceramics, which are widely used in bone regeneration due to their ability to mimic the mineral composition of human bone. Adding small amounts of calcium carbonate as a dopant enhances the material’s solubility and promotes better resorption. This effect is attributed to the induced disorder in the crystalline structure, which enhances osteoblast adhesion, as well as the formation of new bone tissue compared with the pure material [46].

The drawbacks of traditionally used materials, such as low mechanical strength and potential immunogenicity, have prompted the scientific community to explore more efficient alternatives [47]. Among these, hydroxyapatite derived from marine biological resources—such as fish bones and biogenic calcium carbonates (mainly calcite or aragonite)—has shown remarkable versatility [48].

Other marine biological sources, such as corals [49,50], algae [51], and shells [52,53], have been employed in hydroxyapatite production. Notably, calcium carbonate derived from corals stands out for its intrinsic porous structure, which facilitates cellular growth and diffusion while promoting new blood vessel formation, an essential result for successful bone regeneration [54].

Integrating marine-derived materials with growth factors like IGF-1 and BMP-2 presents a novel strategy to enhance bone regeneration by promoting osteogenic differentiation and the formation of new bone tissue. Unlike traditional synthetic and ceramic materials, these biomaterials of marine origin reduce the likelihood of triggering adverse immune reactions [55]. Additionally, the porosity structure of marine calcium carbonate-based scaffolds effectively supports cell integration and tissue formation while degrading with bone regeneration. This is a critical aspect of the long-term success of regenerative therapies [56]. A study reports the effect of calcium carbonate/hydroxyapatite microporous constructs in osteoclastogenesis and shows the regulatory role of Ca^2+^ and L-type voltage-gated ion channels in controlling the induction of bone formation. The extent of induction of bone differentiation is initiated and maintained via the BMP pathway [57].

#### 3.1.2. Magnesium Salts

Magnesium is the third most abundant ion in seawater, after sodium and chloride. It is present in various magnesium minerals as inorganic salts, including magnesium oxide, magnesium sulfate, and magnesium chloride. As an essential element for human health, the body contains approximately 30 grams of magnesium, with 60% of it stored in bone tissue. Magnesium is involved in various biochemical processes and regulates bone cell activity and the mineralization process [58].

Marine sources of magnesium salts, including seawater, brines, and minerals like dolomite and magnesite, offer a sustainable and advantageous alternative, which is extracted by industrial techniques such as evaporation and electrolysis. A notable characteristic of magnesium salts of marine origin is their excellent bioavailability, which improves their absorption and stimulates osteoinduction, promoting the development of new bone tissue [13]. This characteristic makes them advantageous for bone repair therapies, particularly in cases of severe fractures or significant defects [59]. Recent studies demonstrate that, in addition to enhancing cellular activity, marine magnesium salts promote bone healing by regulating immune responses and reducing inflammation. In addition, Wang et al. [60] found that these salts encourage angiogenesis by activating key factors such as vascular endothelial growth factors and angiogenin, which are essential for the vascular development necessary for tissue recovery.

#### 3.1.3. Silica

Silicon is the second most abundant element in the earth’s crust, after oxygen, and accounts for about 27% of its weight. In its pure form, silicon is a semiconductor metal that is not found in nature but can be found in various compounds such as oxides (e.g., quartz, jasper, agate, opal) and silicates (e.g., granite, feldspar, clay, asbestos, mica). The oceans are an important source of silicon, present both as an inorganic element in marine sediments or dissolved in water, as well as a structural component in various marine organisms, such as microorganisms, sponges, and algae, where it plays a crucial role in the formation of protective and supporting structures. Due to its natural abundance and ease of extraction and processing, silicon has become the king material in the microelectronics industry through the development of advanced materials for novel applications, including electronics [61], photonics [62,63,64], and sensor technologies [65,66,67]. In the biomedical field, silicon has garnered particular interest in tissue engineering, where bioactive glasses and silica-based materials have emerged as promising osteoinductive agents [68]. The nanoscale engineering of silica [69,70] has further enabled the production of silica nanoparticles capable of enhancing osteogenic differentiation in human MSCs [71,72].

Diatomite is a valuable biogenic source of silica that originates from fossilized amorphous silica skeletons and photosynthetic algae. These porous materials find applications in controlled drug release [73], nanoparticle production [74], and artificial matrix development [75]. Although the use of silica extracted from marine organisms, such as sponges, is still poorly explored, their skeletons represent an excellent model for tissue engineering applications [76,77].

The combination of silicate-based ceramic materials with different polymers has produced very promising composite scaffolds, which outperform phosphate-based ceramics in biological performance [78].

An innovative example includes diatom-derived biosilica loaded with doxycycline and coated with hydroxybutyl chitosan hydrogel, which has accelerated wound healing and improved hemostasis [78]. In vivo studies in animal models also confirmed the biocompatibility of silica [79].

Designing hierarchical porous scaffolds that mimic the structure and properties of natural bone remains a crucial challenge in bone tissue engineering. In this context, Martins et al. [80] developed three-dimensional porous scaffold composites by combining marine collagen (extracted from codfish skin) with silica-based materials (bioactive glass, diatomite, and sponge-derived biosilica). The findings suggested that integrating silica-based materials into collagen matrices enhances the mechanical and biological properties of biomaterials.

The progress made over the years underscores the potential of composite materials from marine sources as highly effective tools in bone tissue engineering, raising awareness of the biomedical innovations provided by marine-derived resources.

Several studies have shown that soluble silica plays a crucial role in osteogenic differentiation by modulating key molecular pathways [81]. In particular, silica positively regulates the expression of osteogenic markers such as RUNX2, BMP-2, and osteocalcin (OCN) in human dental follicular cells (hDFC). In addition, soluble silica significantly enhances the expression of connexin 43, a protein essential for gap junction communication.

### 3.2. Marine-Derived Organic Materials

#### 3.2.1. Polysaccharides

Marine polysaccharides have been studied for their interesting features, as well as osteogenic potential, biocompatibility, biodegradability, and hydrogel-forming capacity, promoting them as suitable for scaffolding. They also enhance the differentiation of MSC and mineralization of osteoblasts, indicating their potential for bone tissue engineering applications [82]. Through a modulating action of pathways such as the PI3K/Akt and ERK/MAPK signaling cascades, these biomaterials can further support cell survival, proliferation, and differentiation in bone tissue engineering [6].

Among them, alginates of marine origin have been applied to produce hydrogels. They possess low antigenicity, biocompatibility, and the advance to be easily moldable and functionalized in hybrid systems [83]. Alginate scaffold offers a three-dimensional setting in which (i) bioactive molecules can be incorporated to enhance osteoinduction [84], and (ii) stem cells can be encapsulated furnishing the possibility of a co-culture of different cell types to optimize angiogenic and osteogenic potentials [85].

#### 3.2.2. Proteins and Peptides

Marine peptides and proteins also play an active role in bone regeneration mechanisms. For instance, mother-of-pearl and marine collagen, extracted from fish and other marine organisms, are useful as biocompatible support materials that can promote cell attachment, proliferation, and bone growth [86].

In this context, fish are particularly interesting, as collagen is one of the most abundant stromal proteins in their tissues [87], and it can be efficiently extracted from several fish parts [88]. For example, fish skin is one of the most commonly utilized sources for type I collagen extraction. The main sources of marine collagen (skin, scales, bones, muscles, fins, swim bladders, and exoskeletons of marine organisms) are often waste products eliminated by fish distribution centers. This renders it sustainable for a range of applications [89]. Overall, marine peptides, mainly collagen, have received scientific community attention due to their bioactive properties and in promoting bone health. It has been shown to increase bone mineral density, stimulate osteoblastic activity, and provide protective effects against bone degeneration. Moreover, bioactive peptides derived from marine collagen are recognized for enhancing the absorption of calcium and zinc, essential minerals for bone health and the prevention of osteoporosis [90].

#### 3.2.3. Marine-Organism-Derived Cartilage

Cartilage is a gel-like tissue produced by chondrocytes [91] with an extracellular matrix that is abundant in proteoglycans and collagen. Proteoglycans are macromolecules consisting of a protein core with side chains of glycosaminoglycans; the main ones in cartilage include chondroitin sulfate, keratan sulfate, dermatan sulfate, and heparan sulfate [92]. Glycosaminoglycans and collagen play a crucial role in regulating cartilage cell development and function [93]. For this reason, they have been included in foods, pharmaceuticals, and cosmetic products [94].

The increase in demand for cartilage products is due both to an aging population and especially the increase in degenerative and chronic diseases. For instance, chondroitin sulfate and type II collagen derived from animal cartilage are commonly used to alleviate osteoarthritis symptoms [43], as their oral administration has been shown to reduce related inflammatory reactions. In this context, chondroitin sulfate has been demonstrated to be very promising in applications involving medical biomaterials, as it combines with other polymers to form cellular scaffolds for bone regeneration [95], although the degree and manner of bioactivity are contingent on its composition, which may vary depending on the species from which it is derived [96].

For these reasons, the study of the bioactivity of animal cartilage tissues is gaining increasing attention. Particularly, bioactive compounds derived from animal cartilage have multiple bioactivities and have already been widely incorporated into the formulations of numerous products for health purposes.

Commercial production of bioactive compounds mainly involves mammalian and poultry cartilage. However, these sources are associated with zoonotic diseases. As a consequence, cartilage products derived from aquatic organisms have gained increasing attention [97]. This cartilage represents an innovative resource for tissue regeneration due to its unique properties, including high biocompatibility and rich bioactive compounds. Indeed, fish industry waste has emerged as a promising natural source for tissue regeneration, as it contains a wide variety of biomaterials with valuable characteristics for the purpose, including biocompatibility and adequate biodegradability. A recent study has analyzed the properties of the dorsal cartilage of fish with a cartilaginous skeleton, including elasmobranchs (reticulated skate, *Himantura uarnak*, and milk shark *Rhizoprionodon acutus*) and sturgeon (beluga, *Huso huso*) [98]. The findings from the above-mentioned study have determined the efficacy in bone regeneration because they have a skeleton composed entirely of cartilage and, unlike humans, pigs and almost all other animals do not undergo the endochondral ossification process. For this reason, cartilaginous fish, such as sharks and rays, retain their cartilaginous skeleton until adulthood, unlike bony fish [97]. In this context, shark cartilage is the primary source of marine cartilage [98].

#### 3.2.4. Lipids

Marine-derived lipids, especially long-chain polyunsaturated fatty acids, have garnered significant attention for their potential to enhance bone healing and regeneration. Incorporating these lipids into scaffolds, and biomaterials, or using them as supplements in therapeutic treatments are promising approaches to improve bone regeneration outcomes. However, a major challenge with marine-based lipids is ensuring their bioavailability and stability within the body or biomaterials, as they are prone to oxidation [99]. Thus, developing formulations or delivery systems that protect these lipids from oxidative damage is crucial.

Among these lipids, omega-3 fatty acids are essential polyunsaturated fatty acids that play a vital role in various biological functions, such as brain function, cardiovascular health, and inflammation regulation. These fatty acids are abundantly found in marine sources like fish species, including salmon, tuna, and halibut, as well as marine organisms such as algae and krill. The nutritional and therapeutic importance of omega-3 fatty acids has stimulated extensive research into ways to increase their intake, particularly in the context of health and wellness. A recent study conducted by Luján-Amoraga et al. [100] demonstrated that the administration of different concentrations of omega-3 fatty acids can enhance skeletal health, modulating the expression of genes involved in bone cell proliferation, the synthesis of bone components and molecules involved in the interaction, and signaling between bone components or in important cellular processes. The analysis indicated a particular impact on endochondral ossification, as several genes related to cartilage development were also positively modulated. These results offer significant insights into the impact of dietary omega-3 on genes involved in the primary molecular mechanism and cellular processes involved in skeletal development. The regulatory role of fatty acids in the bone restoration process has been a subject of interest in recent years. Recent studies reported the effect of omega-3 polyunsaturated fatty acids on bone fracture healing. Chen et al. [101] demonstrated a significant acceleration of callus formation and fracture healing. Another study evaluated the effect of combining vitamin D3 and omega-3, with results showing that omega-3 can have a positive impact on fracture bonding and arguing that the use of omega-3 PUFA in clinical trials should be increased [102].

Squalene is a lipid predominantly found in shark liver oil. It has been largely studied for its antioxidant, antitumoral, and skin-protective properties. Recently, in vivo studies have demonstrated its role in enhancing the osteoinductive effects of BMP [103].

## 4. Sources of Biomaterials from Marine Environment

In bone repair processes, the potential biological activities of marine derivatives are clearly justified by their chemical composition. Considering that the marine environment is characterized by great biodiversity, great efforts have been made to define the kind of biomaterials obtainable from a specific marine organism and their potential application in bone regenerative medicine. These aspects have been investigated by both in vitro and in vivo studies, as summarized in Table 1 and revised in the next sections.

### 4.1. Seashells

Shells derived from marine organisms are biogenic materials composed mainly of carbonate with dense microstructures. They are used for the treatment of bone defects due to mechanical–chemical and osteoinductive properties, with an improved osteogenic response in bone tissue [121,122].

From the conversion of shells, ceramic materials such as tri-calcium phosphates, hydroxyapatite, and calcium phosphate ceramics are obtained, which are useful biomaterials for bone substitutes and fillers [123].

Bivalve mollusks, such as mussels, show an elongated and asymmetrical shell shape, in contrast to other edible clams, which are generally round or oval. Mussels are the most commonly encountered of these species [124]. The shells of mussels, like those of other bivalve mollusks, consist mainly of calcium carbonate (CaCO_3_). Natural calcium carbonate compounds possess several key characteristics that make them suitable for various medical and health applications. CaCO_3_ is, in fact, an eco-friendly, biocompatible material, a characteristic that limits the risk of immune reactions (especially in cases of implantation) and facilitates integration with bone tissue during the healing process, minimizing the risk of rejection [125]. The use of these shells minimizes waste, reducing the need to extract and process new calcium carbonate. This is an optimal resource (especially when natural populations are managed and harvested in a sustainable manner) able to provide an abundance of calcium carbonate. It is also an economic material, making it a valuable resource for clinical applications such as bone regeneration research and the dental industry [126]. Indeed, their calcareous exoskeletons display a hierarchical composite structure with a textured surface, a complex network of pores, and crystalline formations [127]. This architecture enables the mechanical strength of the shells, promoting cell adhesion and proliferation with a larger surface area for cell interactions [128]. Furthermore, such a structure is able to mimic the architecture of human bone, promoting itself as an excellent and viable option for use in clinical settings [129].

Recent studies have demonstrated the potential role of shell-derived hydroxyapatite (HA) for bone tissue engineering applications. An excellent example that has demonstrated the potential of these materials is that involving the shell of the oyster *Crassostrea gigas*. In this study, a low-temperature one-pot hydrothermal method was developed to convert oyster shell waste into osteoinductive HA micro- and nanoparticles. The particles have proven to be suitable for translational applications in bone regeneration, as they are cytocompatible and can significantly promote osteogenic differentiation of mesenchymal stem cells [126]. In particular, oyster biogene CaCO_3_ particles from shell waste and HA-derived nanoparticles were evaluated for their potential to induce osteogenic differentiation in the murine mesenchymal cell line m17.ASC. After 14 days of treatment with these particles, alkaline phosphatase ALP enzyme activity—a key marker of early osteogenesis—was significantly increased, even without the presence of osteogenic enhancers. Furthermore, Alizarin red staining showed enhanced mineralized matrix deposition, indicating that these materials promote not only the early but also the later stages of the osteogenesis process. These results are in line with previous research, such as the studies by Coringa et al. [130] and Ho et al. [131], which demonstrated the osteoinductive properties of calcium-based materials derived from oyster shells. Similarly, in recent years, research has shown that materials derived from the sea such as shells, corals, and cuttlefish bones can be converted via this method into HA while still retaining their natural architecture that mimics that of humans. When implanted into rat femoral defects, these materials showed bioactivity and osteoconductivity, supporting bone growth and integration. Other innovative methods were successfully applied to produce HA nanoparticles from shells, such as the chemical precipitation with phosphoric acid. The produced nanoparticles were shown to be useful in accelerating bone healing in rabbit mandibular bones, as revealed by histological analysis. Interestingly, in addition to their structural resemblance to bone, seashells also harbor biomineralization proteins critical for bone morphogenesis as the BMPs. Consequently, these materials activate key molecular pathways, including BMP signaling, Wnt signaling, and TGF-β pathways. This highlights their bioactivity and their potential to create a regenerative microenvironment that supports osteogenesis [132].

### 4.2. Coral

Corals are marine invertebrates belonging to the class Anthozoa and the phylum Cnidaria.

The skeleton of these aquatic species has a unique structure and architecture that make it an excellent bone mimetic material for clinical bone regeneration.

In terms of chemical composition, corals consist mainly of CaCO_3_ (up to 99%) and some amino acids and trace elements (organic molecules of the matrix fraction comprising carbohydrates, lipids, and proteins) [133].

Significant similarities were found between the structure of coral and spongy bone, and the in vivo implant was found to be biocompatible. Coral material is organized to form a uniform network of interconnected channels and pores that vary in porosity and volume depending on the species. This is a relevant aspect of bone regeneration.

Indeed, higher porosity and volume have been shown to result in a better response to coral resorption and new bone formation, especially for scaffold applications with pore sizes between 100 μm and 500 μm [134,135,136,137,138,139]. Studies on bone transplant biomaterials from corals have focused mainly on three species: *Acropora* sp., *Goniopora* sp., and *Porties* sp. [115]. The *Cnidaria* coral skeleton, especially in the species mentioned above, shows physical and structural characteristics that guarantee biocompatibility and favorable support for all physiological processes involved in bone repair. These advantageous features of coral scaffolds validate their choice in several clinical applications, as well as in spinal fusion [140,141,142], dental surgery [143,144], maxillofacial surgery [145,146,147,148], and orthopedic [149,150,151]. Successful results in vitro studies show how primary human osteoblasts [152] and a mesenchymal cell line of a mouse (CRL-12424) used coral scaffold as an adhesion surface to support their growth and differentiation [153]. Promising results on successful healing of bone defects have also been obtained in vivo in both cortical and spongy bone defects in dogs, as well as segmental tibial defects in sheep. However, these early successes were later complemented by further findings showing that coral alone is not sufficient to support effective bone healing [147,154], requiring the addition of osteogenic cells to the scaffold [145]. Consequently, to better support bone healing, the use of scaffolds from corals enriched with osteogenic growth factors or cells to improve the osteoinductive properties of the scaffold was evaluated. Nandi et al. evaluated the use of hydrothermally converted coral as a carrier for BMP-2 and IGF-1 to enhance bone formation and osseointegration in a rabbit bone defect model. Their results demonstrated that the natural porous structure of coral scaffolds supports efficient loading and controlled release of growth factors, thereby promoting bone reparation [54]. Other studies showed an improved performance in the bone regeneration process due to the use of enriched coral scaffolds [137]. It has been abundantly shown that bone formation was minimal with the use of coral alone, but healing was significantly improved by adding autologous stromal cells from the bone marrow, as reported for Biocoral^®^ (BioCoral Inc., Inoteb, Saint-Gonnery, France), a commercially available bone substitute derived from purified coral exoskeleton *Porites* [54,137]. To the best of our knowledge, there are different commercially available products with osteoconductive properties obtained from corals, among them are Pro Osteon^®^ (Zimmer Biomet, Irvine, CA, USA) and CoreBone^®^ (Corebone, Misgav, HaZafon, Isdrael).

### 4.3. Mussels’ Byssus

The byssus is an anchoring structure present in all bivalve mollusks that is produced as early as post-larval settlement. In mussels, specifically, it also persists in the adult forms [155], which ensure a firm grip on the substrate [156] with a semipermanent attachment technique.

The byssus consists of numerous byssal threads, which in the case of *Mytilus galloprovincialis*, are almost entirely composed of protein and produced by a specific organ, the mussel’s foot [157]. Each byssal thread can be divided into morphologically and compositionally distinct sections, i.e., the proximal portion, which connects to the soft tissue, and the distal portion, which in turn, together with the adhesive disk or plaque, enables anchoring [158,159].

Mussels secrete mussel adhesive protein (MAP) from their byssal threads and adhesive plaques, which is biocompatible, biodegradable, and nontoxic [160]. More than ten foot proteins, including collagens (preCol-NG, preCol-C, and preCol-D), thread matrix proteins (Ptmp and Tmp), and mussel foot proteins (Mfp-1 to Mfp-6), have been isolated and identified from marine mussels, highlighting the complexity and functionality of these materials [161].

Among the reported molecules, MAP has been successfully identified as a coating agent for implants. To date, titanium implants were modified by mussel foot proteins (Mefp-1 and Mefp-2), and the biological evaluation by in vitro and in vivo models reported a greater bone regeneration with respect to the nonmodified implants [160]. Similar results were obtained by the evaluation of attachment, proliferation, and osteogenic differentiation of human adipose tissue-derived stem cells on a MAP-functionalized three-dimensional scaffold of polycaprolactone/poly(lactic-co-glycolic acid) [162].

### 4.4. Jellyfish

Jellyfish (or medusae) are a group of freely swimming organisms belonging to the phylum Cnidaria. Commonly, jellyfish are targets of study due to their toxic proteins. However, jellyfish are a valuable marine resource in the field of bone regeneration. Collagen is the most abundant protein in living organisms and plays a key role in structural function in bones and cartilage. Gelatin is produced by partial hydrolysis of collagen and subsequent heat treatment. Two types of gelatin are generally obtained, indicated as type A (acid hydrolysis) and type B (alkaline hydrolysis) according to the type of method used [163]. Therefore, collagen is the precursor of gelatin, which consists of a mixture of single or multiple-filament polypeptides, each containing about 50–1000 amino acids. The amino acid composition of gelatin is predominantly rich in residues of glycine, proline, and 4-hydroxyproline. Minor constituents are glutamic acid, alanine, arginine, aspartic acid, lysine, serine, leucine, valine, phenylalanine, threonine, isoleucine, hydroxylysine, methionine, histidine, and tyrosine. The percentage amount of each amino acid varies, especially for minor components, depending on the source of raw material and processing technique.

Jellyfish gelatin contains moisture, crude protein, crude lipid, and crude ash [164,165]. Promising results for regenerative medicine were obtained from the study by Rigogliuso et al. [119], which demonstrated the efficacy of an injectable marine hydrogel obtained from collagen native to the jellyfish *Rhizostoma pulmo* with hydroxyphenyl propionic acid (HPA)-functionalized marine gelatin. A biocompatible hydrogel able to enzymatically cross-link using horseradish peroxidase (HPR) and H_2_O_2_ favoring the placement of cells inside. Indeed, several scientific results obtained support the biochemical properties of marine collagen on the differentiation of chondrocytes, thus encouraging its use in cartilage engineering and tissue repair [166,167]. In the field of cartilage regeneration research, the focus is on biomaterials that mimic physiological structures. For this reason, collagen fibrous structures and/or combined products such as hydrogels are proving to be useful tools as scaffolding and support the engineering of cartilage tissue. In vitro cartilage regeneration requires that the material chosen as structure (e.g., collagen) must ensure interaction with the cell and subsequent production of extracellular matrix. Literature studies show how collagen-based porous scaffolds have been applied for concurrent differentiation of mesenchymal stem cells (MSCs) in vitro and have been widely used for in vivo chondroma transplantation. Furthermore, it is interesting to note that collagen extracted from the jellyfish *Rhopilema esculentum* is similar to human collagen II despite the evolutionary distance. In addition, this type of collagen scaffold has been shown to support the chondrogenic differentiation pattern better than conventional scaffolds of porcine collagen I [168,169]. Among the reasons that encourage the choice of jellyfish collagen, there is at first the large evolutionary distance which reduces the risk of disease transmission in clinical practice with respect to bovine or porcine-derived biomaterials. In order to obtain an alternative and safer biomaterial an acid-soluble collagen has been extracted from jellyfish. MTT assay results show that jellyfish collagen exhibited greater cellular vitality than other naturally derived biomaterials, including bovine collagen, gelatin, hyaluronic acid, and glucan [170,171]. Secondly, the jellyfish-derived hydrogels have a softer consistency than fibrous scaffolds and can promote cell differentiation of MSC toward the chondrogenic line [172,173]. In addition, the high water content of hydrogels promotes the diffusion of nutrients, wastes, and signaling molecules [174].

## 5. Marine Microbial Polymers

The marine microbial biosphere, due to its enormous biodiversity, is an almost unlimited source of novel biomolecules that can help meet the growing demand for novel bioactive natural molecules for medical and nonmedical applications. In particular, microbes from extreme environments synthesize polymers, including exopolysaccharides (EPS), with unique chemical and physical characteristics, which allow them to survive in these unusual conditions [175,176]. These biopolymers have shown diverse biological activities ranging from antimicrobial to antioxidant. Furthermore, in the context of eco-sustainable production, microalgae as autotrophic photosynthetic organisms are playing an increasingly important role in blue biotechnology. Hence, we explored the potentialities in bone tissue regeneration of EPS produced by marine bacteria and marine microalgae extracts with a focus on their activities on regenerative molecular patterns.

### 5.1. Bacterial Exopolysaccharides

Bacterial exopolysaccharides (EPSs) are water-soluble polymers composed differing in monosaccharides constituents and molecular weight (MW) [177], and based on their chemical composition, they can be classified as homopolysaccharides or heteropolysaccharides (including pentoses, such as D-arabinose, D-ribose, and D-xylose; hexoses, such as D-glucose, D-galactose, D-mannose, D-allose, L-rhamnose, and L-fucose; amino sugars, such as D-glucosamine and D-galactosamine; or uronic acids, such as D-glucuronic and D-galacturonic acids) [178], as well as organic or inorganic substituents, such as sulfate, phosphate, acetic acid, succinic acid, and pyruvic acid, conferring to EPSs physicochemical properties. EPSs are the major component of the bacterial biofilm matrix that play several roles, such as adhesion to abiotic or biotic surfaces, reserve for water and nutrients, and protection against physical, chemical, and biological agents [179].

These biopolymers are gaining enormous impact in the biotechnological field, serving as antibiofilm, anti-inflammatory, antimicrobial agents, or in bioremediation as heavy metal chelating agents. Moreover, EPSs attract great interest in regenerative medicine due to their structure, which mimics polysaccharides found in human cells’ extracellular matrix [180]. Some terrestrial bacteria such as *Streptococcus zooepidemicus*, *Acetobacter xylinum, Gluconacetobacter, Pseudomonas, Sarcina, Azobacter, Rhizobium,* and *Agrobacterium* species [181] produce polysaccharides similar to the hyaluronic acid or collagens composing the matrix of articular cartilage of the human body [182]. The use of these EPSs could improve lubrication, cell migration [183], and hydration in epithelia and promote the adhesion and growth of human cells [184]. Moreover, cellulose-rich EPSs exhibit moderate antibacterial activity against common bone-infecting bacteria, such as *Staphylococcus aureus* and *Escherichia coli* [185].

Marine ecosystems, including those of extreme environments such as deep and shallow hydrothermal vents in the Arctic and Antarctic Sea, offer a great and almost untapped resource for novel biopolymers that could be exploited in different fields, including regenerative medicine.

The *Vibrio diabolicus* isolated from *Alveniella pompeiana* inhabiting deep hydrothermal vents produced an EPS (HE800 EPS) composed of glucuronic acid and hexosamine (N-acetyl glucosamine and N-acetyl galactosamine) that conferred similar properties of hyaluronic acid [186,187]. This polymer contributed to restoring the bone tissue of rats after 15 days of treatment. The activity of HE800 EPS was related to the increasing tissue vascularization mediating interactions between bone cells and matrix. Moreover, this polymer inhibits the secretion of matrix metalloproteinases (MMPs) (i.e., gelatinase A MMP-2) and stromelysin 1 (MMP-3) in fibroblasts, reducing the inflammatory responses [186].

*Alteromonas infernus* was isolated from a sample of fluid collected among a dense population of *Riftia pachyptila*. This bacterium produces an EPS named GY785 composed of uronic acid (galacturonic and glucuronic acid), galactose, and glucose; moreover, the presence of the sulfate group increased the viability and proliferation of the chondrocytes [188]. Interestingly, the GY785 EPS was treated to obtain derivates with lower molecular weight that exhibited the ability to inhibit tissue breakdown and inflammation interfering with complement cascade and secretion of matrix metalloproteinases by inflammatory cytokines such as IL-1β and TNF-α.

The mannose-rich EPS-B315, produced by the marine thermophilic *Bacillus licheniformis* B315, was thermostable (up to 80 °C) and noncytotoxic (up to 300 µg/ml); moreover, it possessed several biological activities, making it a strong candidate for biomedical applications [189,190,191]. The EPS B3-15 exhibited antibiofilm against Gram-negative bacteria (*Pseudomonas aeruginosa* and *Klebsiella pneumoniae*) and Gram-positive bacteria (*Staphylococcus aureus* and *Streptococcus pneumoniae*) on abiotic surfaces (e.g., polystyrene, PVC medical devices, and contact lenses), as well as biotic surfaces (e.g., human nasal epithelial cells) [191]. Moreover, this biopolymer showed anti-inflammatory activities reducing inflammation in RAW 264.7 cells and suggesting its potential use in contrasting both bacterial infections and inflammation [190,191,192]. Mannose-rich polysaccharides interact with Toll-like receptors (TLRs) to activate host immunity [193], specifically by activating TLR-2 and TLR-4, increasing IL-6, and downregulating IL-8. Therefore, EPS-B315 having a similar monosaccharide composition could activate macrophages by binding its sugar units to carbohydrate receptors on their surface. The interaction with macrophages could lead to the production of osteogenic factors that accelerate bone healing offering new perspectives in regenerative medicine [194,195]. Despite the advantages of EPSs produced by thermophilic bacteria, including rapid growth, high yields, and excellent thermostability, to our knowledge, only a few exopolysaccharides (Table 2) have been used for osteogenic purposes, indicating promising targets for future research.

### 5.2. Microalgae

The interest in microalgae as a source of novel natural compounds is linked to their ability to grow sustainably, reducing CO_2_ emissions and meeting the principles of the blue economy [196]. Several microalgal species hold commercial importance, including *Spirulina*, *Chlorella*, *Haematococcus*, *Dunaliella*, *Botryococcus*, *Phaeodactylum*, *Porphyridium*, *Chaetoceros*, *Crypthecodinium*, *Isochrysis*, *Nannochloris*, *Nitzschia*, *Schizochytrium*, *Tetraselmis*, and *Skeletonema* [197]. Moreover, many of these species can produce high-value molecules with antioxidant, anti-inflammatory, antiaging, and immunostimulant properties that find application in nutraceutical, cosmeceutical, pharmaceutical, and medicine fields [198]. Marine microalgae have been evaluated as a source of a plethora of different compounds including fatty acids useful for the production of biofuel or as nutrients for animals and humans, carbohydrates, protein, and vitamins for animal feeds [199,200].

Innovative applications of products derived from different species of microalgae include the mineralization and osteogenesis of bone. The ethanolic extract of the *Chlorophyta, Skeletonema costatum,* and *Tetraselmis striata* CTP4 have been reported for their activity in the mineralization of repair action on *Sparus aurata* bone and inducing the differentiation of osteoblastic cells from *Danio rerio* [201]. These extracts can upregulate the expression of the genes related to bone formation, bone modulation, and those involved in antioxidant activity. Differently, the aqueous extract of *Arthospira platensis* increased the expression of the β-catenin gene in human amniotic mesenchymal stem cells [202].

Microalgae such as *Chlorella pyrenoidosa*, *Nannochloropsis oceanica, Arthospira platensis*, and *Dunaliella salina* are widely recognized as a promising source of biomass, which provides significant quantities of proteins able to chelate calcium and contrast osteoporosis in the zebrafish model, reducing the effects of dexamethasone-induced osteoporosis [203]. Moreover, the peptide (NOP) derived from microalgae *Nannochloropsis oculata* was shown to stimulate human osteoblast (MG-63) and murine mesenchymal stem (D1) cells by increasing alkaline phosphatase, osteocalcin, and bone mineralization. The presence of NOP NOP-activated molecular cascade enhances the MAP phosphorylation of kinases (p-ERK, p-JNK, p-p38) and therefore the Smads (p-Smad1/5/8) expression [204].

The whole biomasses from *Chlorella*, *Chlamydomonas reinhardtii*, and *Arthospira platensis* were used as scaffolds for tissue regeneration. Indeed, they were able to stimulate the growth, adhesion, and proliferation of cells [196] (Table 2).

**Table 2:** Polymers derived from marine microorganisms useful for bone regeneration and their molecular target.

Polymer	Microorganism	Composition	Osteogenesis Molecular Target	References
HE800 EPS	*Vibrio diabolicus*	*N-acetyl glucosamine and N-acetyl galactosamine*	*IL-1β* induction, the secretion of matrix metalloproteinases, stromelysin 1	[186]
EPS GY785	*Alteromonas infernus*	Galacturonic and glucuronic acid) and galactose and glucose	Complement cascade cytokines inhibition of IL-1β and TNF-α	[188]
EPS-B315	*Bacillus licheniformis B3-15*	Mannose–glucose	Activating TLR-2 and TLR-4 downregulating IL-8	[192]
Ethanolic extract	*Skeletonema costatum*	n.d.	Upregulation of genes involved in bone synthesis and remodeling (p7, col1a1a, oc1, and oc2; acp5a	[201]
Ethanolic extract	*Tetraselmis striata*	n.d.	Upregulation of genes involved in bone synthesis and remodeling (p7, col1a1a, oc1, and oc2; acp5a Antioxidant gene (cat, sod1)	[201]
Aqueous extract	*Arthospira platensis*	n.d.	Upregulation of β- catenin in human amniotic mesenchymal stem cells	[202]
NOP	*Nannochloropsis oculata*	Peptide	stimulate human osteoblast (MG-63) upregulate p-Smad1/5/8	[204]
Biomass	*Chlamydomonas reinhardtii*	n.d.	Scaffolds increase the adhesion and growth of human cells	[196]

n.d. = not determined.

In addition to biomedical applications, *Chlorella vulgaris* has been used to heal wounds, exhibiting anti-inflammatory and antibacterial activity against pathogens such as *Klebsiella pneumoniae*, *Enterococcus faecalis*, and *Escherichia coli* [196,205]. Its extracts promote collagen deposition and reduce fibroblasts, benefiting tissue regeneration. Similarly, *Chlamydomonas reinhardtii* has been employed to create photosynthetic scaffolds for oxygen delivery and tissue repair, showing anti-inflammatory properties when tested in vivo [205].

## 6. Innovative Tools for Bone Tissue Engineering: Phage Display and Marine-Derived Peptides

To further enhance the applicability of marine resources such as algae and other marine, phage display technology can be employed to identify and optimize peptides with high specificity for bone-related targets. Phage display (PD) technology is an innovative approach in which functional exogenous peptides are exposed on the capsid surface of a bacteriophage as a fusion product with endogenous proteins of the phage coat. Recently, several research groups used PD technology to select tissue-specific peptides able to stimulate cell adhesion, proliferation, and differentiation, improving osteochondral regeneration [206,207].

**Figure 3:**
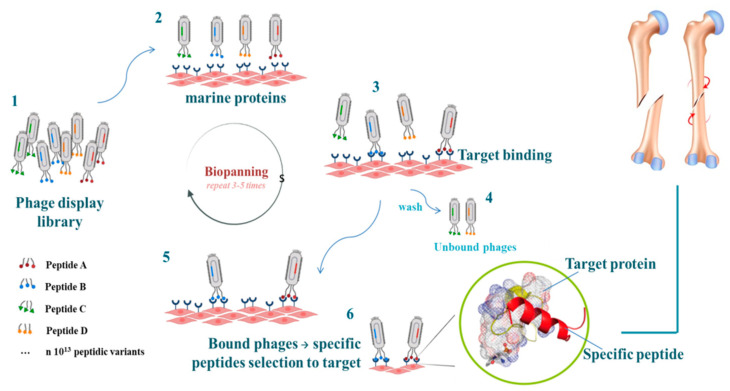
Biopanning via phage display technology allows the identification of novel peptide sequences selected from marine materials. (Created partially with BioRender.com, Toronto, ON, Canada, www.biorender.com.)

Marine adhesive proteins, such as those derived from mussels and barnacles, are resources for designing peptides with adhesive properties, as well as for human cells. These proteins exhibit exceptional binding capabilities in aqueous environments, mainly due to specific amino acid residues such as dihydroxyphenylalanine (DOPA) detected in mussel proteins. As shown in Figure 3, phage display technology allows the identification of novel peptide sequences selected from these natural adhesives, optimizing their potential for bone regeneration and other biomedical applications [208,209].

The combination of marine-derived peptides and phage display technology enables the development of highly specific and functionalized biomaterials.

This approach opens the door to multifunctional peptides with immense potential from marine resources in promoting sustainable and innovative regenerative medicine.

## 7. Conclusions

The exploration of traditional and innovative materials such as calcium carbonate, magnesium salts, silica, polysaccharides, and bioactive peptides could overcome the limitations of conventional therapies. These materials, in fact, on the one hand, activate and modulate osteogenic pathways involved in bone growth, such as BMP/Smad and Wnt/β-catenin, and on the other hand, can be incorporated into composite scaffolds for the application of advanced technologies. Furthermore, the use of phage display technology could improve the identification of novel sequences that enhance their regenerative contribution. By exploiting these presented advances, it is possible to design next-generation biomaterials that mimic the natural bone environment and that can be used to improve bone health, promoting a paradigm shift in regenerative medicine. Moreover, the use of biomaterials of marine origin also represents an opportunity to promote the circular economy, reducing waste and providing a concrete response to the demand for raw materials for biomedical applications.

## Figures and Tables

**Table 1:** Marine-derived biomaterials involved in bone regeneration.

Marine Source	Marine-Derived Biomaterial	Chemical Category	Use/Biological Activity	References
Diatom skeleton	Diatomite, natural diatomaceoussediment that can stably release Silicon ions	Mineral	Osteochondral repair scaffold promotes osteogenic differentiation of BMSCs (bone marrow mesenchymal stem cells) and enhances proliferation and maturation of chondrocytes	[74,104,105,106,107]
Red algae *Lithothamnion corallioides*	Aquamin is a natural, multimineral, rich in calcium, magnesium, and 72 other trace minerals	Mineral	Aquamin supplement promotes increased mineralization in osteoblast cell culture	[108,109]
Sponge skeleton	Biosilica is abiocompatible, natural inorganic polymer formed by an enzymatic silicatein-mediated reaction in siliceous sponges to build up their inorganic skeleton	Mineral	Microspheres containing β-TCP and silica, and β-TCP and silicatein, induce the mineralization of human osteoblast-like cells (SaOS-2) in vitro;capability to support cell adhesion and proliferation	[80,110,111,112]
Seashell	Mussel shell hydroxyapatite(combined with chitosan and gentamicin sulfate antibiotic)	Mineral	Composite with biocompatibility and antibacterial activity (enhanced osteoblast cell proliferation)	[113]
Seashell	Aragonite is acowrie-shell-derived powder	Mineral	Biocompatibility with human stem cells; useful carrier/scaffold for bone tissue engineering and raw material for 3D-printed orthopedic devices	[114]
Coral skeletons	Corals, mainly the species *Acropora* sp., *Goniopora* sp., and *Porties* sp.	Mineral matrix fraction (peptide, lipid, carbohydrates)	Extraction and use of coral matrix proteins (osteoinduction);laboratory-grown coral materials for bone;framework for hard tissue engineering with new and superior properties; augmented coral skeleton;permeation with synthetic materials biomolecules and mineral ions	[115]
Mussel’s byssus	3,4-dihydroxyphenyl-l-alanine (Dopa) catecholic amino acid in mussel adhesive proteins	Adhesive protein amino acid	In vitro 3,4-dihydroxyphenyl-l-alanine (Dopa)-coated plates enhance osteogenic differentiation of human mesenchymal stem cells’functional coating with an integrin-targeting sequence to improve cell adhesion by mussel byssus-inspired surface chemistry enhancing osteogenic activity in zirconia dental implants	[116,117,118]
Jellyfish	Injectable marine hydrogel obtained from collagen native to the jellyfish *Rhizostoma pulmo* with hydroxyphenyl propionic acid (HPA)–functionalized marine gelatin	Protein lipid	Biocompatible hydrogel able to enzymatically cross-link using horseradish peroxidase (HPR) and H_2_ O_2_ favoring the placement of cells inside	[119]
Jellyfish	Collagen scaffold	Protein (collagen)	In vivo, jellyfish collagen scaffolds showed a long-term anti-inflammatory macrophage response and an optimal vascularization pattern within their implant beds, showing excellent biocompatibility and (bone) tissue regenerative properties	[120]

## References

[1] Berendsen A.D., Olsen B.R. (2015). Bone Development. Bone.

[2] Zaidi M. (2007). Skeletal Remodeling in Health and Disease. Nat. Med..

[3] Javaheri B., Caetano-Silva S.P., Kanakis I., Bou-Gharios G., Pitsillides A.A. (2018). The Chondro-Osseous Continuum: Is It Possible to Unlock the Potential Assigned Within?. Front. Bioeng. Biotechnol..

[4] Kronenberg H.M. (2003). Developmental Regulation of the Growth Plate. Nature.

[5] Trompet D., Melis S., Chagin A.S., Maes C. (2024). Skeletal Stem and Progenitor Cells in Bone Development and Repair. J. Bone Miner. Res..

[6] Vermeulen S., Tahmasebi Birgani Z., Habibovic P. (2022). Biomaterial-Induced Pathway Modulation for Bone Regeneration. Biomaterials.

[7] Ashfaq R., Kovács A., Berkó S., Budai-Szűcs M. (2024). Developments in Alloplastic Bone Grafts and Barrier Membrane Biomaterials for Periodontal Guided Tissue and Bone Regeneration Therapy. Int. J. Mol. Sci..

[8] Bauer T.W., Muschler G.F. (2000). Bone Graft Materials: An Overview of the Basic Science. Clin. Orthop. Relat. Res.®.

[9] Alonzo M., Alvarez Primo F., Anil Kumar S., Mudloff J.A., Dominguez E., Fregoso G., Ortiz N., Weiss W.M., Joddar B. (2021). Bone Tissue Engineering Techniques, Advances, and Scaffolds for Treatment of Bone Defects. Curr. Opin. Biomed. Eng..

[10] Girón J., Kerstner E., Medeiros T., Oliveira L., Machado G.M., Malfatti C.F., Pranke P. (2021). Biomaterials for Bone Regeneration: An Orthopedic and Dentistry Overview. Braz. J. Med. Biol. Res..

[11] Oryan A., Alidadi S., Moshiri A., Maffulli N. (2014). Bone Regenerative Medicine: Classic Options, Novel Strategies, and Future Directions. J. Orthop. Surg. Res..

[12] Ho-Shui-Ling A., Bolander J., Rustom L.E., Johnson A.W., Luyten F.P., Picart C. (2018). Bone Regeneration Strategies: Engineered Scaffolds, Bioactive Molecules and Stem Cells Current Stage and Future Perspectives. Biomaterials.

[13] Wang H., Li X., Xuan M., Yang R., Zhang J., Chang J. (2024). Marine Biomaterials for Sustainable Bone Regeneration. Giant.

[14] Marsell R., Einhorn T.A. (2011). The Biology of Fracture Healing. Injury.

[15] Chen D., Zhao M., Mundy G.R. (2004). Bone Morphogenetic Proteins. Growth Factors.

[16] Boyce B.F., Xing L. (2008). Functions of RANKL/RANK/OPG in Bone Modeling and Remodeling. Arch. Biochem. Biophys..

[17] Lin G.L., Hankenson K.D. (2011). Integration of BMP, Wnt, and Notch Signaling Pathways in Osteoblast Differentiation. J. Cell. Biochem..

[18] Čolnik M., Primožič M., Knez Ž., Leitgeb M. (2016). Use of Non-Conventional Cell Disruption Method for Extraction of Proteins from Black Yeasts. Front. Bioeng. Biotechnol..

[19] Nauth A., Schemitsch E., Norris B., Nollin Z., Watson J.T. (2018). Critical-Size Bone Defects: Is There a Consensus for Diagnosis and Treatment?. J. Orthop. Trauma.

[20] Hutchings G., Moncrieff L., Dompe C., Janowicz K., Sibiak R., Bryja A., Jankowski M., Mozdziak P., Bukowska D., Antosik P. (2020). Bone Regeneration, Reconstruction and Use of Osteogenic Cells; from Basic Knowledge, Animal Models to Clinical Trials. J. Clin. Med..

[21] García-Gareta E., Coathup M.J., Blunn G.W. (2015). Osteoinduction of Bone Grafting Materials for Bone Repair and Regeneration. Bone.

[22] Veronesi F., Maglio M., Brogini S., Fini M. (2020). In Vivo Studies on Osteoinduction: A Systematic Review on Animal Models, Implant Site, and Type and Postimplantation Investigation. J. Biomed. Mater. Res. Part A.

[23] Albrektsson T., Johansson C. (2001). Osteoinduction, Osteoconduction and Osseointegration. Eur. Spine J..

[24] Solheim E. (1998). Osteoinduction by Demineralised Bone. Int. Orthop. SICOT.

[25] Schmidmaier G., Wildemann B., Ostapowicz D., Kandziora F., Stange R., Haas N.P., Raschke M. (2004). Long-Term Effects of Local Growth Factor (IGF-I and TGF-Β1) Treatment on Fracture Healing: A Safety Study for Using Growth Factors. J. Orthop. Res..

[26] Barradas A.M., Yuan H., van Blitterswijk C., Habibovic P. (2010). Osteoinductive Biomaterials: Current Knowledge of Properties, Experimental Models and Biological Mechanisms. eCM.

[27] Mehta M., Schmidt-Bleek K., Duda G.N., Mooney D.J. (2012). Biomaterial Delivery of Morphogens to Mimic the Natural Healing Cascade in Bone. Adv. Drug Deliv. Rev..

[28] Kirker-Head C.A. (2000). Potential Applications and Delivery Strategies for Bone Morphogenetic Proteins. Adv. Drug Deliv. Rev..

[29] Polini A., Pisignano D., Parodi M., Quarto R., Scaglione S. (2011). Osteoinduction of Human Mesenchymal Stem Cells by Bioactive Composite Scaffolds without Supplemental Osteogenic Growth Factors. PLoS ONE.

[30] Kübler N.R. (1997). Osteoinduction and -reparation. Mund Kiefer Gesichtschir.

[31] Yuan H., de Bruijn J.D., Zhang X., van Blitterswijk C.A., de Groot K. (2001). Bone Induction by Porous Glass Ceramic Made from Bioglass^®^ (45S5). J. Biomed. Mater. Res..

[32] Yan W.-Q., Nakamura T., Kawanabe K., Nishigochi S., Oka M., Kokubo T. (1997). Apatite Layer-Coated Titanium for Use as Bone Bonding Implants. Biomaterials.

[33] Nygren H., Ilver L., Malmberg P. (2016). Mineralization at Titanium Surfaces Is a Two-Step Process. J. Funct. Biomater..

[34] Wróbel E., Witkowska-Zimny M., Przybylski J. (2010). Biological Mechanisms of Implant Osseointegration. Ortop. Traumatol. Rehabil..

[35] Ragone V., Canciani E., Arosio M., Olimpo M., Piras L.A., von Degerfeld M.M., Augusti D., D’Ambrosi R., Dellavia C. (2020). In Vivo Osseointegration of a Randomized Trabecular Titanium Structure Obtained by an Additive Manufacturing Technique. J. Mater. Sci. Mater. Med..

[36] Fabbro M.D., Taschieri S., Canciani E., Addis A., Musto F., Weinstein R., Dellavia C. (2017). Osseointegration of Titanium Implants With Different Rough Surfaces: A Histologic and Histomorphometric Study in an Adult Minipig Model. Implant Dent..

[37] Lagopati N., Pippa N., Gatou M.-A., Papadopoulou-Fermeli N., Gorgoulis V.G., Gazouli M., Pavlatou E.A. (2023). Marine-Originated Materials and Their Potential Use in Biomedicine. Appl. Sci..

[38] Singh H., Parida A., Debbarma K., Ray D.P., Banerjee P. (2020). Common Marine Organisms: A Novel Source of Medicinal Compounds. IJBS.

[39] Wang Y., Chen L., Wang Y., Wang X., Qian D., Yan J., Sun Z., Cui P., Yu L., Wu J. (2023). Marine Biomaterials in Biomedical Nano/Micro-Systems. J. Nanobiotechnol..

[40] Kiuru P., D’Auria M.V., Muller C.D., Tammela P., Vuorela H., Yli-Kauhaluoma J. (2014). Exploring Marine Resources for Bioactive Compounds. Planta Medica.

[41] Poblete-Castro I., Hoffmann S.-L., Becker J., Wittmann C. (2020). Cascaded Valorization of Seaweed Using Microbial Cell Factories. Curr. Opin. Biotechnol..

[42] Cao H., Zeng Y., Yuan X., Wang J.K., Tay C.Y. (2025). Waste-to-Resource: Extraction and Transformation of Aquatic Biomaterials for Regenerative Medicine. Biomater. Adv..

[43] Ben-Nissan B., Choi A.H., Green D.W., Choi A.H., Ben-Nissan B. (2019). Marine Derived Biomaterials for Bone Regeneration and Tissue Engineering: Learning from Nature. Marine-Derived Biomaterials for Tissue Engineering Applications.

[44] Foran E., Weiner S., Fine M. (2013). Biogenic Fish-Gut Calcium Carbonate Is a Stable Amorphous Phase in the Gilt-Head Seabream, Sparus Aurata. Sci. Rep..

[45] Qi C., Zhu Y.-J., Lu B.-Q., Zhao X.-Y., Zhao J., Chen F., Wu J. (2014). ATP-Stabilized Amorphous Calcium Carbonate Nanospheres and Their Application in Protein Adsorption. Small.

[46] Šupová M. (2014). Isolation and Preparation of Nanoscale Bioapatites from Natural Sources: A Review. J. Nanosci. Nanotechnol..

[47] Bharadwaz A., Jayasuriya A.C. (2020). Recent Trends in the Application of Widely Used Natural and Synthetic Polymer Nanocomposites in Bone Tissue Regeneration. Mater. Sci. Eng. C.

[48] Akram M., Ahmed R., Shakir I., Ibrahim W.A.W., Hussain R. (2014). Extracting Hydroxyapatite and Its Precursors from Natural Resources. J. Mater. Sci..

[49] Sivakumar M., Kumar T.S.S., Shantha K.L., Rao K.P. (1996). Development of Hydroxyapatite Derived from Indian Coral. Biomaterials.

[50] Jinawath S., Polchai D., Yoshimura M. (2002). Low-Temperature, Hydrothermal Transformation of Aragonite to Hydroxyapatite. Mater. Sci. Eng. C.

[51] Walsh P.J., Buchanan F.J., Dring M., Maggs C., Bell S., Walker G.M. (2008). Low-Pressure Synthesis and Characterisation of Hydroxyapatite Derived from Mineralise Red Algae. Chem. Eng. J..

[52] Kumar G.S., Girija E.K., Venkatesh M., Karunakaran G., Kolesnikov E., Kuznetsov D. (2017). One Step Method to Synthesize Flower-like Hydroxyapatite Architecture Using Mussel Shell Bio-Waste as a Calcium Source. Ceram. Int..

[53] Pal A., Maity S., Chabri S., Bera S., Chowdhury A.R., Das M., Sinha A. (2017). Mechanochemical Synthesis of Nanocrystalline Hydroxyapatite from Mercenaria Clam Shells and Phosphoric Acid. Biomed. Phys. Eng. Express.

[54] Nandi S.K., Kundu B., Mukherjee J., Mahato A., Datta S., Balla V.K. (2015). Converted Marine Coral Hydroxyapatite Implants with Growth Factors: In Vivo Bone Regeneration. Mater. Sci. Eng. C.

[55] Lalzawmliana V., Anand A., Mukherjee P., Chaudhuri S., Kundu B., Nandi S.K., Thakur N.L. (2019). Marine Organisms as a Source of Natural Matrix for Bone Tissue Engineering. Ceram. Int..

[56] Neto A.S., Ferreira J.M.F. (2018). Synthetic and Marine-Derived Porous Scaffolds for Bone Tissue Engineering. Materials.

[57] Klar R.M., Duarte R., Dix-Peek T., Dickens C., Ferretti C., Ripamonti U. (2013). Calcium Ions and Osteoclastogenesis Initiate the Induction of Bone Formation by Coral-Derived Macroporous Constructs. J. Cell. Mol. Med..

[58] Zhou H., Liang B., Jiang H., Deng Z., Yu K. (2021). Magnesium-Based Biomaterials as Emerging Agents for Bone Repair and Regeneration: From Mechanism to Application. J. Magnes. Alloys.

[59] Bavya Devi K., Nandi S.K., Roy M. (2019). Magnesium Silicate Bioceramics for Bone Regeneration: A Review. J. Indian Inst. Sci..

[60] Wang J.-L., Xu J.-K., Hopkins C., Chow D.H.-K., Qin L. (2020). Biodegradable Magnesium-Based Implants in Orthopedics—A General Review and Perspectives. Adv. Sci..

[61] Zwanenburg F.A., Dzurak A.S., Morello A., Simmons M.Y., Hollenberg L.C.L., Klimeck G., Rogge S., Coppersmith S.N., Eriksson M.A. (2013). Silicon Quantum Electronics. Rev. Mod. Phys..

[62] Morganti D., Leonardi A.A., Lo Faro M.J., Leonardi G., Salvato G., Fazio B., Musumeci P., Livreri P., Conoci S., Neri G. (2021). Ultrathin Silicon Nanowires for Optical and Electrical Nitrogen Dioxide Detection. Nanomaterials.

[63] Lo Faro M.J., Ruello G., Leonardi A.A., Morganti D., Irrera A., Priolo F., Gigan S., Volpe G., Fazio B. (2021). Visualization of Directional Beaming of Weakly Localized Raman from a Random Network of Silicon Nanowires. Adv. Sci..

[64] Leonardi A.A., Sciuto E.L., Lo Faro M.J., Morganti D., Midiri A., Spinella C., Conoci S., Irrera A., Fazio B. (2022). Molecular Fingerprinting of the Omicron Variant Genome of SARS-CoV-2 by SERS Spectroscopy. Nanomaterials.

[65] Harraz F.A. (2014). Porous Silicon Chemical Sensors and Biosensors: A Review. Sens. Actuators B Chem..

[66] Leonardi A.A., Battaglia R., Morganti D., Faro M.J.L., Fazio B., De Pascali C., Francioso L., Palazzo G., Mallardi A., Purrello M. (2021). A Novel Silicon Platform for Selective Isolation, Quantification, and Molecular Analysis of Small Extracellular Vesicles. Int. J. Nanomed..

[67] Morganti D., Faro M.J.L., Leonardi A.A., Fazio B., Conoci S., Irrera A. (2022). Luminescent Silicon Nanowires as Novel Sensor for Environmental Air Quality Control. Sensors.

[68] Habibovic P., de Groot K. (2007). Osteoinductive Biomaterials—Properties and Relevance in Bone Repair. J. Tissue Eng. Regen. Med..

[69] Pedraza A.J., Fowlkes J.D., Guan Y.-F. (2003). Surface Nanostructuring of Silicon. Appl. Phys. A.

[70] Huang Z., Geyer N., Werner P., de Boor J., Gösele U. (2011). Metal-Assisted Chemical Etching of Silicon: A Review. Adv. Mater..

[71] Yang X., Li Y., Liu X., Huang Q., He W., Zhang R., Feng Q., Benayahu D. (2016). The Stimulatory Effect of Silica Nanoparticles on Osteogenic Differentiation of Human Mesenchymal Stem Cells. Biomed. Mater..

[72] Gaharwar A.K., Mihaila S.M., Swami A., Patel A., Sant S., Reis R.L., Marques A.P., Gomes M.E., Khademhosseini A. (2013). Bioactive Silicate Nanoplatelets for Osteogenic Differentiation of Human Mesenchymal Stem Cells. Adv. Mater..

[73] Aw M.S., Simovic S., Yu Y., Addai-Mensah J., Losic D. (2012). Porous Silica Microshells from Diatoms as Biocarrier for Drug Delivery Applications. Powder Technol..

[74] Le T.D.H., Bonani W., Speranza G., Sglavo V., Ceccato R., Maniglio D., Motta A., Migliaresi C. (2016). Processing and Characterization of Diatom Nanoparticles and Microparticles as Potential Source of Silicon for Bone Tissue Engineering. Mater. Sci. Eng. C.

[75] Losic D., Mitchell J.G., Voelcker N.H. (2009). Diatomaceous Lessons in Nanotechnology and Advanced Materials. Adv. Mater..

[76] Martins E., Rapp H.T., Xavier J.R., Diogo G.S., Reis R.L., Silva T.H. (2021). Macro and Microstructural Characteristics of North Atlantic Deep-Sea Sponges as Bioinspired Models for Tissue Engineering Scaffolding. Front. Mar. Sci..

[77] Ahmadipour M., Mohammadi H., Pang A.L., Arjmand M., Ayode Otitoju T., Okoye P.U., Rajitha B. (2022). A Review: Silicate Ceramic-Polymer Composite Scaffold for Bone Tissue Engineering. Int. J. Polym. Mater. Polym. Biomater..

[78] Rozan H.E., Wu G., Zhou Z., Li Q., Sharaf M., Chen X. (2022). The Complex Hydrogel Based on Diatom Biosilica and Hydroxybutyl Chitosan for Wound Healing. Colloids Surf. B Biointerfaces.

[79] Wiens M., Wang X., Schloßmacher U., Lieberwirth I., Glasser G., Ushijima H., Schröder H.C., Müller W.E.G. (2010). Osteogenic Potential of Biosilica on Human Osteoblast-Like (SaOS-2) Cells. Calcif. Tissue Int..

[80] Martins E., Diogo G.S., Pires R., Reis R.L., Silva T.H. (2022). 3D Biocomposites Comprising Marine Collagen and Silica-Based Materials Inspired on the Composition of Marine Sponge Skeletons Envisaging Bone Tissue Regeneration. Mar. Drugs.

[81] Aslam B., Augustyniak A., Clarke S.A., McMahon H. (2023). Development of a Novel Marine-Derived Tricomposite Biomaterial for Bone Regeneration. Mar. Drugs.

[82] Senni K., Pereira J., Gueniche F., Delbarre-Ladrat C., Sinquin C., Ratiskol J., Godeau G., Fischer A.-M., Helley D., Colliec-Jouault S. (2011). Marine Polysaccharides: A Source of Bioactive Molecules for Cell Therapy and Tissue Engineering. Mar. Drugs.

[83] Kolambkar Y.M., Dupont K.M., Boerckel J.D., Huebsch N., Mooney D.J., Hutmacher D.W., Guldberg R.E. (2011). An Alginate-Based Hybrid System for Growth Factor Delivery in the Functional Repair of Large Bone Defects. Biomaterials.

[84] Barre A., Naudot M., Colin F., Sevestre H., Collet L., Devauchelle B., Lack S., Marolleau J.-P., Le Ricousse S. (2020). An Alginate-Based Hydrogel with a High Angiogenic Capacity and a High Osteogenic Potential. BioRes. Open Access.

[85] Xu N., Peng X.-L., Li H.-R., Liu J.-X., Cheng J.-S.-Y., Qi X.-Y., Ye S.-J., Gong H.-L., Zhao X.-H., Yu J. (2021). Marine-Derived Collagen as Biomaterials for Human Health. Front. Nutr..

[86] Muralidharan N., Jeya Shakila R., Sukumar D., Jeyasekaran G. (2013). Skin, Bone and Muscle Collagen Extraction from the Trash Fish, Leather Jacket (Odonus Niger) and Their Characterization. J. Food Sci. Technol..

[87] Hou Y., Shavandi A., Carne A., Bekhit A.A., Ng T.B., Cheung R.C.F., Bekhit A.E.A. (2016). Marine Shells: Potential Opportunities for Extraction of Functional and Health-Promoting Materials. Crit. Rev. Environ. Sci. Technol..

[88] Meng K., Chen L., Xia G., shen X. (2021). Effects of Zinc Sulfate and Zinc Lactate on the Properties of Tilapia (*Oreochromis Niloticus*) Skin Collagen Peptide Chelate Zinc. Food Chem..

[89] Witten P.E., Huysseune A., Hall B.K. (2010). A Practical Approach for the Identification of the Many Cartilaginous Tissues in Teleost Fish. J. Appl. Ichthyol..

[90] Vázquez J.A., Rodríguez-Amado I., Montemayor M.I., Fraguas J., González M.D.P., Murado M.A. (2013). Chondroitin Sulfate, Hyaluronic Acid and Chitin/Chitosan Production Using Marine Waste Sources: Characteristics, Applications and Eco-Friendly Processes: A Review. Mar. Drugs.

[91] Knudson C.B., Knudson W. (2001). Cartilage Proteoglycans. Semin. Cell Dev. Biol..

[92] Volpi N. (2019). Chondroitin Sulfate Safety and Quality. Molecules.

[93] Li W., Ura K., Takagi Y. (2022). Industrial Application of Fish Cartilaginous Tissues. Curr. Res. Food Sci..

[94] Varghese S., Hwang N.S., Canver A.C., Theprungsirikul P., Lin D.W., Elisseeff J. (2008). Chondroitin Sulfate Based Niches for Chondrogenic Differentiation of Mesenchymal Stem Cells. Matrix Biol..

[95] Ashhurst D.E. (2004). The Cartilaginous Skeleton of an Elasmobranch Fish Does Not Heal. Matrix Biol..

[96] Zamani A., Khajavi M., Nazarpak M.H., Solouk A., Atef M. (2024). Preliminary Evaluation of Fish Cartilage as a Promising Biomaterial in Cartilage Tissue Engineering. Ann. Anat.—Anat. Anz..

[97] Seidel R., Blumer M., Pechriggl E.-J., Lyons K., Hall B.K., Fratzl P., Weaver J.C., Dean M.N. (2017). Calcified Cartilage or Bone? Collagens in the Tessellated Endoskeletons of Cartilaginous Fish (Sharks and Rays). J. Struct. Biol..

[98] Dean M.N., Summers A.P. (2006). Mineralized Cartilage in the Skeleton of Chondrichthyan Fishes. Zoology.

[99] Aubourg S.P. (2001). Fluorescence Study of the Pro-oxidant Effect of Free Fatty Acids on Marine Lipids. J. Sci. Food Agric..

[100] Luján-Amoraga L., Delgado-Martín B., Lourenço-Marques C., Gavaia P.J., Bravo J., Bandarra N.M., Dominguez D., Izquierdo M.S., Pousão-Ferreira P., Ribeiro L. (2024). Exploring Omega-3′s Impact on the Expression of Bone-Related Genes in Meagre (*Argyrosomus Regius*). Biomolecules.

[101] Chen Y., Cao H., Sun D., Lin C., Wang L., Huang M., Jiang H., Zhang Z., Jin D., Zhang B. (2017). Endogenous Production of N-3 Polyunsaturated Fatty Acids Promotes Fracture Healing in Mice. J. Healthc. Eng..

[102] Banu J., Bhattacharya A., Rahman M., Kang J.X., Fernandes G. (2010). Endogenously Produced N-3 Fatty Acids Protect against Ovariectomy Induced Bone Loss in Fat-1 Transgenic Mice. J. Bone Miner. Metab..

[103] Kawakami T., Kawai T., Takei N., Kise T., Eda S., Urist M.R. (1997). Evaluation of Heterotopic Bone Formation Induced by Squalane and Bone Morphogenetic Protein Composite. Clin. Orthop. Relat. Res. (1976–2007).

[104] Deng C., Qin C., Li Z., Lu L., Tong Y., Yuan J., Yin F., Cheng Y., Wu C. (2024). Diatomite-Incorporated Hierarchical Scaffolds for Osteochondral Regeneration. Bioact. Mater..

[105] Tamburaci S., Kimna C., Tihminlioglu F. (2019). Bioactive Diatomite and POSS Silica Cage Reinforced Chitosan/Na-Carboxymethyl Cellulose Polyelectrolyte Scaffolds for Hard Tissue Regeneration. Mater. Sci. Eng. C.

[106] Min K.H., Kim D.H., Youn S., Pack S.P. (2024). Biomimetic Diatom Biosilica and Its Potential for Biomedical Applications and Prospects: A Review. Int. J. Mol. Sci..

[107] Olăreț E., Dinescu S., Dobranici A.-E., Ginghină R.-E., Voicu G., Mihăilescu M., Curti F., Banciu D.D., Sava B., Amarie S. (2024). Osteoblast Responsive Biosilica-Enriched Gelatin Microfibrillar Microenvironments. Biomater. Adv..

[108] O’Gorman D.M., Tierney C.M., Brennan O., O’Brien F.J. (2012). The Marine-Derived, Multi-Mineral Formula, Aquamin, Enhances Mineralisation of Osteoblast Cells In Vitro. Phytother. Res..

[109] Widaa A., Brennan O., O’Gorman D.M., O’Brien F.J. (2014). The Osteogenic Potential of the Marine-Derived Multi-Mineral Formula Aquamin Is Enhanced by the Presence of Vitamin D. Phytother. Res..

[110] Wang S., Wang X., Draenert F.G., Albert O., Schröder H.C., Mailänder V., Mitov G., Müller W.E.G. (2014). Bioactive and Biodegradable Silica Biomaterial for Bone Regeneration. Bone.

[111] Wang X., Schröder H.C., Wiens M., Schloßmacher U., Müller W.E.G., Becerro M.A., Uriz M.J., Maldonado M., Turon X. (2012). Chapter Five—Biosilica: Molecular Biology, Biochemistry and Function in Demosponges as Well as Its Applied Aspects for Tissue Engineering. Advances in Marine Biology.

[112] Müller W.E., Neufurth M., Wang S., Schröder H.C., Wang X. (2024). The Physiological Inorganic Polymers Biosilica and Polyphosphate as Key Drivers for Biomedical Materials in Regenerative Nanomedicine. Int. J. Nanomed..

[113] Sathiyavimal S., Vasantharaj S., Mattheos N., Pugazhendhi A., Subbalekha K. (2024). Mussel Shell-Derived Biogenic Hydroxyapatite as Reinforcement on Chitosan-Loaded Gentamicin Composite for Antibacterial Activity and Bone Regeneration. Int. J. Biol. Macromol..

[114] Akilal N., Lemaire F., Bercu N.B., Sayen S., Gangloff S.C., Khelfaoui Y., Rammal H., Kerdjoudj H. (2019). Cowries Derived Aragonite as Raw Biomaterials for Bone Regenerative Medicine. Mater. Sci. Eng. C.

[115] Green D.W., Ben-Nissan B., Yoon K.S., Milthorpe B., Jung H.-S. (2017). Natural and Synthetic Coral Biomineralization for Human Bone Revitalization. Trends Biotechnol..

[116] La W.-G., Shin J.-Y., Bhang S.H., Jin M., Yoon H.H., Noh S.-S., Im G.-I., Kim C.-S., Kim B.-S. (2013). Culture on a 3,4-Dihydroxy-l-Phenylalanine-Coated Surface Promotes the Osteogenic Differentiation of Human Mesenchymal Stem Cells. Tissue Eng. Part A.

[117] Zhang Q., Wu S., Sun Y., Ru Yie K.H., Zhuang J., Liu T., Si W., Zhang Y., Liu Z., Xiong L. (2024). Mussel Byssus-Inspired Dual-Functionalization of Zirconia Dental Implants for Improved Bone Integration. Mater. Today Bio.

[118] Gao P., Zhang Q., Sun Y., Cheng H., Wu S., Zhang Y., Si W., Sun H., Sun N., Yang J. (2025). Synergistic Catecholamine and Coordination Chemistry for Enhanced Bioactivity and Secondary Grafting Activity of Zirconia Dental Implants. Colloids Surf. B Biointerfaces.

[119] Rigogliuso S., Salamone M., Barbarino E., Barbarino M., Nicosia A., Ghersi G. (2020). Production of Injectable Marine Collagen-Based Hydrogel for the Maintenance of Differentiated Chondrocytes in Tissue Engineering Applications. Int. J. Mol. Sci..

[120] Flaig I., Radenković M., Najman S., Pröhl A., Jung O., Barbeck M. (2020). In Vivo Analysis of the Biocompatibility and Immune Response of Jellyfish Collagen Scaffolds and Its Suitability for Bone Regeneration. Int. J. Mol. Sci..

[121] Atlan G., Balmain N., Berland S., Vidal B., Lopez É. (1997). Reconstruction of Human Maxillary Defects with Nacre Powder: Histological Evidence for Bone Regeneration. Comptes Rendus L’académie Sci.-Ser. III-Sci. Vie.

[122] Alakpa E.V., Burgess K.E.V., Chung P., Riehle M.O., Gadegaard N., Dalby M.J., Cusack M. (2017). Nacre Topography Produces Higher Crystallinity in Bone than Chemically Induced Osteogenesis. ACS Nano.

[123] Sun F., Zhou H., Lee J. (2011). Various Preparation Methods of Highly Porous Hydroxyapatite/Polymer Nanoscale Biocomposites for Bone Regeneration. Acta Biomater..

[124] Oktar F.N., Unal S., Gunduz O., Nissan B.B., Macha I.J., Akyol S., Duta L., Ekren N., Altan E., Yetmez M. (2023). Marine-Derived Bioceramics for Orthopedic, Reconstructive and Dental Surgery Applications. J. Aust. Ceram. Soc..

[125] Muntean F.L., Olariu I., Marian D., Olariu T., Petrescu E.L., Olariu T., Drăghici G.A. (2024). Hydroxyapatite from Mollusk Shells: Characteristics, Production, and Potential Applications in Dentistry. Dent. J..

[126] Fernández-Penas R., Verdugo-Escamilla C., Triunfo C., Gärtner S., D’Urso A., Oltolina F., Follenzi A., Maoloni G., Cölfen H., Falini G. (2023). A Sustainable One-Pot Method to Transform Seashell Waste Calcium Carbonate to Osteoinductive Hydroxyapatite Micro-Nanoparticles. J. Mater. Chem. B.

[127] Cheng M., Liu M., Chang L., Liu Q., Wang C., Hu L., Zhang Z., Ding W., Chen L., Guo S. (2023). Overview of Structure, Function and Integrated Utilization of Marine Shell. Sci. Total Environ..

[128] Azarian M.H., Sutapun W. (2022). Biogenic Calcium Carbonate Derived from Waste Shells for Advanced Material Applications: A Review. Front. Mater..

[129] Zhang X., Vecchio K.S. (2013). Conversion of Natural Marine Skeletons as Scaffolds for Bone Tissue Engineering. Front. Mater. Sci..

[130] Coringa R., de Sousa E.M., Botelho J.N., Diniz R.S., de Sá J.C., da Cruz M.C.F.N., Paschoal M.A.B., Gonçalves L.M. (2018). Bone Substitute Made from a Brazilian Oyster Shell Functions as a Fast Stimulator for Bone-Forming Cells in an Animal Model. PLoS ONE.

[131] Ho W.-F., Lee M.-H., Thomas J.L., Li J.-A., Wu S.-C., Hsu H.-C., Lin H.-Y. (2021). Porous Biphasic Calcium Phosphate Granules from Oyster Shell Promote the Differentiation of Induced Pluripotent Stem Cells. Int. J. Mol. Sci..

[132] Green D.W., Padula M., Santos J., Chou J., Milthorpe B., Ben-Nissan B. (2013). A New Role for Marine Skeletal Proteins in Regenerative Orthopaedics. Key Eng. Mater..

[133] Falini G., Fermani S., Goffredo S. (2015). Coral Biomineralization: A Focus on Intra-Skeletal Organic Matrix and Calcification. Semin. Cell Dev. Biol..

[134] Abramovitch-Gottlib L., Geresh S., Vago R. (2006). Biofabricated Marine Hydrozoan: A Bioactive Crystalline Material Promoting Ossification of Mesenchymal Stem Cells. Tissue Eng..

[135] Geiger F., Lorenz H., Xu W., Szalay K., Kasten P., Claes L., Augat P., Richter W. (2007). VEGF Producing Bone Marrow Stromal Cells (BMSC) Enhance Vascularization and Resorption of a Natural Coral Bone Substitute. Bone.

[136] Green D., Walsh D., Mann S., Oreffo R.O.C. (2002). The Potential of Biomimesis in Bone Tissue Engineering: Lessons from the Design and Synthesis of Invertebrate Skeletons. Bone.

[137] Mygind T., Stiehler M., Baatrup A., Li H., Zou X., Flyvbjerg A., Kassem M., Bünger C. (2007). Mesenchymal Stem Cell Ingrowth and Differentiation on Coralline Hydroxyapatite Scaffolds. Biomaterials.

[138] Shors E.C. (1999). Coralline Bone Graft Substitutes. Orthop. Clin..

[139] Wiesmann H.P., Joos U., Meyer U. (2004). Biological and Biophysical Principles in Extracorporal Bone Tissue Engineering: Part II. Int. J. Oral Maxillofac. Surg..

[140] Coughlin M.J., Grimes J.S., Kennedy M.P. (2006). Coralline Hydroxyapatite Bone Graft Substitute in Hindfoot Surgery. Foot Ankle Int..

[141] Griesshaber E., Schmahl W.W., Neuser R., Pettke T., Blüm M., Mutterlose J., Brand U. (2007). Crystallographic Texture and Microstructure of Terebratulide Brachiopod Shell Calcite: An Optimized Materials Design with Hierarchical Architecture. Am. Mineral..

[142] Im K., Park J.H., Kim K.N., Kim K.M., Choi S.H., Kim C.K., Lee Y.K. (2005). Organic-Inorganic Hybrids of Hydroxyapatite with Chitosan. Key Eng. Mater..

[143] Lahaye M., Robic A. (2007). Structure and Functional Properties of Ulvan, a Polysaccharide from Green Seaweeds. Biomacromolecules.

[144] Martina M., Subramanyam G., Weaver J.C., Hutmacher D.W., Morse D.E., Valiyaveettil S. (2005). Developing Macroporous Bicontinuous Materials as Scaffolds for Tissue Engineering. Biomaterials.

[145] Chen P.-Y., Lin A.Y.M., Lin Y.-S., Seki Y., Stokes A.G., Peyras J., Olevsky E.A., Meyers M.A., McKittrick J. (2008). Structure and Mechanical Properties of Selected Biological Materials. J. Mech. Behav. Biomed. Mater..

[146] Green D., Howard D., Yang X., Kelly M., Oreffo R. (2003). Natural Marine Sponge Fiber Skeleton: A Biomimetic Scaffold for Human Osteoprogenitor Cell Attachment, Growth, and Differentiation. Tissue Eng..

[147] Laza A.L., Jaber M., Miehé-Brendlé J., Demais H., Le Deit H., Delmotte L., Vidal L. (2007). Green Nanocomposites: Synthesis and Characterization. J. Nanosci. Nanotechnol..

[148] Oliveira J.M., Grech J.M.R., Leonor I.B., Mano J.F., Reis R.L. (2007). Calcium-Phosphate Derived from Mineralized Algae for Bone Tissue Engineering Applications. Mater. Lett..

[149] Bächle M., Hübner U., Kohal R.J., Han J.S., Wiedmann-Al-Ahmad M. (2006). Structure and *in Vitro* Cytocompatibility of the Gastropod Shell of *Helix Pomatia*. Tissue Cell.

[150] Hou R., Chen F., Yang Y., Cheng X., Gao Z., Yang H.O., Wu W., Mao T. (2007). Comparative Study between Coral-Mesenchymal Stem Cells-rhBMP-2 Composite and Auto-Bone-Graft in Rabbit Critical-Sized Cranial Defect Model. J. Biomed. Mater. Res. Part A.

[151] Kujala S., Raatikainen T., Ryhänen J., Kaarela O., Jalovaara P. (2002). Composite Implant of Native Bovine Bone Morphogenetic Protein (BMP) and Biocoral in the Treatment of Scaphoid Nonunions—A Preliminary Study. Scand. J. Surg..

[152] Doherty M.J., Schlag G., Schwarz N., Mollan R.A.B., Nolan P.C., Wilson D.J. (1994). Biocompatibility of Xenogeneic Bone, Commercially Available Coral, a Bioceramic and Tissue Sealant for Human Osteoblasts. Biomaterials.

[153] Gravel M., Vago R., Tabrizian M. (2006). Use of Natural Coralline Biomaterials As Reinforcing and Gas-Forming Agent for Developing Novel Hybrid Biomatrices: Microarchitectural and Mechanical Studies. Tissue Eng..

[154] Galea G., Kopman D., Graham B.J.M. (1998). Supply and Demand of Bone Allograft for Revision Hip Surgery in Scotland. J. Bone Jt. Surg. Br. Vol..

[155] Waite J.H., Qin X.-X., Coyne K.J. (1998). The Peculiar Collagens of Mussel Byssus. Matrix Biol..

[156] Suhre M.H., Gertz M., Steegborn C., Scheibel T. (2014). Structural and Functional Features of a Collagen-Binding Matrix Protein from the Mussel Byssus. Nat. Commun..

[157] Hagenau A., Scheidt H.A., Serpell L., Huster D., Scheibel T. (2009). Structural Analysis of Proteinaceous Components in Byssal Threads of the Mussel Mytilus Galloprovincialis. Macromol. Biosci..

[158] Babarro J.M.F., Reiriz M.J.F. (2010). Secretion of Byssal Threads in Mytilus Galloprovincialis: Quantitative and Qualitative Values after Spawning Stress. J. Comp. Physiol. B.

[159] Impellitteri F., Yunko K., Calabrese G., Porretti M., Martyniuk V., Gnatyshyna L., Nava V., Potortì A.G., Piccione G., Di Bella G. (2024). Chlorpromazine’s Impact on *Mytilus Galloprovincialis*: A Multi-Faceted Investigation. Chemosphere.

[160] Yin D., Komasa S., Yoshimine S., Sekino T., Okazaki J. (2019). Effect of Mussel Adhesive Protein Coating on Osteogenesis in Vitro and Osteointegration in Vivo to Alkali-Treated Titanium with Nanonetwork Structures. Int. J. Nanomed..

[161] Waite J.H. (2017). Mussel Adhesion–Essential Footwork. J. Exp. Biol..

[162] Hong J.M., Kim B.J., Shim J.-H., Kang K.S., Kim K.-J., Rhie J.W., Cha H.J., Cho D.-W. (2012). Enhancement of Bone Regeneration through Facile Surface Functionalization of Solid Freeform Fabrication-Based Three-Dimensional Scaffolds Using Mussel Adhesive Proteins. Acta Biomater..

[163] Mariod A.A., Fadul H. (2013). Gelatin, Source, Extraction and Industrial Applications. Acta Sci. Pol. Technol. Aliment..

[164] Lueyot A., Rungsardthong V., Vatanyoopaisarn S., Hutangura P., Wonganu B., Wongsa-Ngasri P., Charoenlappanit S., Roytrakul S., Thumthanaruk B. (2021). Influence of Collagen and Some Proteins on Gel Properties of Jellyfish Gelatin. PLoS ONE.

[165] Coppola D., Oliviero M., Vitale G.A., Lauritano C., D’Ambra I., Iannace S., de Pascale D. (2020). Marine Collagen from Alternative and Sustainable Sources: Extraction, Processing and Applications. Mar. Drugs.

[166] Sewing J., Klinger M., Notbohm H. (2017). Jellyfish Collagen Matrices Conserve the Chondrogenic Phenotype in Two- and Three-Dimensional Collagen Matrices. J. Tissue Eng. Regen. Med..

[167] Hoyer B., Bernhardt A., Lode A., Heinemann S., Sewing J., Klinger M., Notbohm H., Gelinsky M. (2014). Jellyfish Collagen Scaffolds for Cartilage Tissue Engineering. Acta Biomater..

[168] Pustlauk W., Paul B., Brueggemeier S., Gelinsky M., Bernhardt A. (2017). Modulation of Chondrogenic Differentiation of Human Mesenchymal Stem Cells in Jellyfish Collagen Scaffolds by Cell Density and Culture Medium. J. Tissue Eng. Regen. Med..

[169] Pustlauk W., Paul B., Gelinsky M., Bernhardt A. (2016). Jellyfish Collagen and Alginate: Combined Marine Materials for Superior Chondrogenesis of hMSC. Mater. Sci. Eng. C.

[170] Song E., Yeon Kim S., Chun T., Byun H.-J., Lee Y.M. (2006). Collagen Scaffolds Derived from a Marine Source and Their Biocompatibility. Biomaterials.

[171] Addad S., Exposito J.-Y., Faye C., Ricard-Blum S., Lethias C. (2011). Isolation, Characterization and Biological Evaluation of Jellyfish Collagen for Use in Biomedical Applications. Mar. Drugs.

[172] Steward A.J., Wagner D.R., Kelly D.J. (2013). The Pericellular Environment Regulates Cytoskeletal Development and the Differentiation of Mesenchymal Stem Cells and Determines Their Response to Hydrostatic Pressure. Eur. Cell Mater..

[173] Mousavi S.J., Doweidar M.H. (2015). Role of Mechanical Cues in Cell Differentiation and Proliferation: A 3D Numerical Model. PLoS ONE.

[174] Li Z., Gunn J., Chen M.-H., Cooper A., Zhang M. (2008). On-Site Alginate Gelation for Enhanced Cell Proliferation and Uniform Distribution in Porous Scaffolds. J. Biomed. Mater. Res. Part A.

[175] Casillo A., Lanzetta R., Parrilli M., Corsaro M.M. (2018). Exopolysaccharides from Marine and Marine Extremophilic Bacteria: Structures, Properties, Ecological Roles and Applications. Mar. Drugs.

[176] Nwodo U.U., Green E., Okoh A.I. (2012). Bacterial Exopolysaccharides: Functionality and Prospects. Int. J. Mol. Sci..

[177] Finore I., Di Donato P., Mastascusa V., Nicolaus B., Poli A. (2014). Fermentation Technologies for the Optimization of Marine Microbial Exopolysaccharide Production. Mar. Drugs.

[178] Freitas F., Torres C.A.V., Reis M.A.M. (2017). Engineering Aspects of Microbial Exopolysaccharide Production. Bioresour. Technol..

[179] Möckl L. (2020). The Emerging Role of the Mammalian Glycocalyx in Functional Membrane Organization and Immune System Regulation. Front. Cell Dev. Biol..

[180] Torgbo S., Sukyai P. (2018). Bacterial Cellulose-Based Scaffold Materials for Bone Tissue Engineering. Appl. Mater. Today.

[181] Witzler M., Büchner D., Shoushrah S.H., Babczyk P., Baranova J., Witzleben S., Tobiasch E., Schulze M. (2019). Polysaccharide-Based Systems for Targeted Stem Cell Differentiation and Bone Regeneration. Biomolecules.

[182] Litwiniuk M., Krejner A., Speyrer M.S., Gauto A.R., Grzela T. (2016). Hyaluronic Acid in Inflammation and Tissue Regeneration. Wounds.

[183] DeVore D.P., Hughes E., Scott J.B. (1994). Effectiveness of Injectable Filler Materials for Smoothing Wrinkle Lines and Depressed Scars. Med. Prog. Technol..

[184] Shi Q., Li Y., Sun J., Zhang H., Chen L., Chen B., Yang H., Wang Z. (2012). The Osteogenesis of Bacterial Cellulose Scaffold Loaded with Bone Morphogenetic Protein-2. Biomaterials.

[185] Zhang H.Y., Yan X.J., Jiang Y., Cong J. (2011). Development and Characteristic of Bacterial Cellulose for Antimicrobial Wound Dressing. Adv. Mater. Res..

[186] Bagnol R., Grijpma D., Eglin D., Moriarty T.F. (2022). The Production and Application of Bacterial Exopolysaccharides as Biomaterials for Bone Regeneration. Carbohydr. Polym..

[187] Zanchetta P., Lagarde N., Guezennec J. (2003). Systemic Effects on Bone Healing of a New Hyaluronic Acid-Like Bacterial Exopolysaccharide. Calcif. Tissue Int..

[188] Ruiz Velasco C., Baud’Huin M., Sinquin C., Maillasson M., Heymann D., Colliec-Jouault S., Padrines M. (2011). Effects of a Sulfated Exopolysaccharide Produced by Altermonas Infernus on Bone Biology. Glycobiology.

[189] Caccamo M.T., Gugliandolo C., Zammuto V., Magazù S. (2020). Thermal Properties of an Exopolysaccharide Produced by a Marine Thermotolerant *Bacillus Licheniformis* by ATR-FTIR Spectroscopy. Int. J. Biol. Macromol..

[190] Zammuto V., Spanò A., Agostino E., Macrì A., De Pasquale C., Ferlazzo G., Rizzo M.G., Nicolò M.S., Guglielmino S., Gugliandolo C. (2023). Anti-Bacterial Adhesion on Abiotic and Biotic Surfaces of the Exopolysaccharide from the Marine Bacillus Licheniformis B3-15. Mar. Drugs.

[191] Zammuto V., Agostino E., Macrì A., Spanò A., Grillo E., Nicolò M.S., Gugliandolo C. (2023). Synergistic Antibiofilm Effects of Exopolymers Produced by the Marine, Thermotolerant Bacillus Licheniformis B3-15 and Their Potential Medical Applications. J. Mar. Sci. Eng..

[192] Rizzo M.G., Zammuto V., Spanò A., Gugliandolo C., Calabrese G., Guglielmino S. (2024). Anti-Inflammatory Effects in LPS-Induced Macrophages and Antibiofilm Activity of the Mannose-Rich Exopolysaccharide Produced by Bacillus Licheniformis B3-15. Heliyon.

[193] Shao B.-M., Dai H., Xu W., Lin Z.-B., Gao X.-M. (2004). Immune Receptors for Polysaccharides from *Ganoderma Lucidum*. Biochem. Biophys. Res. Commun..

[194] Hao S., Meng J., Zhang Y., Liu J., Nie X., Wu F., Yang Y., Wang C., Gu N., Xu H. (2017). Macrophage Phenotypic Mechanomodulation of Enhancing Bone Regeneration by Superparamagnetic Scaffold upon Magnetization. Biomaterials.

[195] Raguénès G., Peres A., Ruimy R., Pignet P., Christen R., Loaec M., Rougeaux H., Barbier G., Guezennec J. (1997). Alteromonas Infernus Sp. Nov., a New Polysaccharide-producing Bacterium Isolated from a Deep-sea Hydrothermal Vent. J. Appl. Microbiol..

[196] Arrieta Payares L.M., Gutiérrez Púa L.D.C., Di Mare Pareja L.A., Paredes Méndez S.C., Paredes Méndez V.N. (2023). Microalgae Applications to Bone Repairing Processes: A Review. ACS Biomater. Sci. Eng..

[197] Raposo M.F. (2013). de J.; De Morais, R.M.S.C.; Bernardo de Morais, A.M.M. Bioactivity and Applications of Sulphated Polysaccharides from Marine Microalgae. Mar. Drugs.

[198] Merlo S., Gabarrell Durany X., Pedroso Tonon A., Rossi S. (2021). Marine Microalgae Contribution to Sustainable Development. Water.

[199] Kumar V., Kashyap M., Gautam S., Shukla P., Joshi K.B., Vinayak V. (2018). Fast Fourier Infrared Spectroscopy to Characterize the Biochemical Composition in Diatoms. J. Biosci..

[200] Markou G., Arapoglou D., Eliopoulos C., Balafoutis A., Taddeo R., Panara A., Thomaidis N. (2019). Cultivation and Safety Aspects of *Arthrospira Platensis* (Spirulina) Grown with Struvite Recovered from Anaerobic Digestion Plant as Phosphorus Source. Algal Res..

[201] Carletti A., Rosa J.T., Pes K., Borges I., Santos T., Barreira L., Varela J., Pereira H., Cancela M.L., Gavaia P.J. (2023). The Osteogenic and Mineralogenic Potential of the Microalgae Skeletonema Costatum and Tetraselmis Striata CTP4 in Fish Models. Cell. Mol. Life Sci..

[202] Moradi F., Hadavi M., Aghamaali M.R., Fallah S.F. (2024). Arthrospira Platensis and Gracilaria Gracilis Algae Extracts as Biological Inducers for Human Amniotic Mesenchymal Stem Cells (hAMSC). J. Appl. Phycol..

[203] Yu H., Chen Y., Zhu J. (2022). Osteogenic Activities of Four Calcium-Chelating Microalgae Peptides. J. Sci. Food Agric..

[204] Nguyen M.H.T. (2012). Osteoblastic Differentiation Effects of a Novel Peptide from Biodiesel By-Products of Microalgae Nannochloropsis Oculata on MG-63 and D1 Cells. Ph.D. Dissertation.

[205] de Melo R.G., de Andrade A.F., Bezerra R.P., Viana Marques D.d.A., da Silva V.A., Paz S.T., de Lima Filho J.L., Porto A.L.F. (2019). Hydrogel-Based Chlorella Vulgaris Extracts: A New Topical Formulation for Wound Healing Treatment. J. Appl. Phycol..

[206] Zambrano-Mila M.S., Blacio K.E.S., Vispo N.S. (2020). Peptide Phage Display: Molecular Principles and Biomedical Applications. Ther. Innov. Regul. Sci..

[207] Rizzo M.G., Palermo N., D’Amora U., Oddo S., Guglielmino S.P.P., Conoci S., Szychlinska M.A., Calabrese G. (2022). Multipotential Role of Growth Factor Mimetic Peptides for Osteochondral Tissue Engineering. Int. J. Mol. Sci..

[208] Rizzo M.G., Plano L.M.D., Palermo N., Franco D., Nicolò M., Sciuto E.L., Calabrese G., Oddo S., Conoci S., Guglielmino S.P.P. (2023). A Novel Serum-Based Diagnosis of Alzheimer’s Disease Using an Advanced Phage-Based Biochip. Adv. Sci..

[209] Rizzo M.G., Carnazza S., De Plano L.M., Franco D., Nicolò M.S., Zammuto V., Petralia S., Calabrese G., Gugliandolo C., Conoci S. (2021). Rapid Detection of Bacterial Pathogens in Blood through Engineered Phages-Beads and Integrated Real-Time PCR into MicroChip. Sens. Actuators B Chem..

